# Better together: biomimetic nanomedicines for high performance tumor therapy

**DOI:** 10.3762/bjnano.16.92

**Published:** 2025-08-05

**Authors:** Imran Shair Mohammad, Gizem Kursunluoglu, Anup Kumar Patel, Hafiz Muhammad Ishaq, Cansu Umran Tunc, Dilek Kanarya, Mubashar Rehman, Omer Aydin, Yin Lifang

**Affiliations:** 1 Department of Radiology, City of Hope National Medical Center, 1500 East Duarte Rd., Duarte, California 91010, USA; 2 Nanothera Lab, Drug Application and Research Center (ERFARMA), Erciyes University, 38039, Kayseri, Turkey; 3 Faculty of Veterinary and Animal Sciences, Muhammad Nawaz Shareef University of Agriculture, Multan 66000, Pakistan; 4 Utah Center for Nanomedicine, University of Utah, Salt Lake City, UT, 84112, USA; 5 Department of Pharmacy, Quaid-i-Azam University, Islamabad 45320, Pakistan; 6 Department of Biomedical Engineering, Erciyes University, 38039, Kayseri, Turkey; 7 Nanotechnology Research and Application Center (ERNAM), Erciyes University, Kayseri 38039, Turkey; 8 Clinical Engineering Research and Implementation Center (ERKAM), Erciyes University, Kayseri 38040, Turkey; 9 Department of Pharmaceutics, School of Pharmacy, China Pharmaceutical University, Nanjing 211198, PR China

**Keywords:** biomimetic nanoparticles, homotypic binding, nanomaterials, targeted drug delivery, tumor therapy

## Abstract

The emergence of nanotechnology offers a promising avenue for enhancing cancer treatment outcomes. In this context, biomimetic nanoparticles have emerged as an exciting frontier in the field of biomedicine. These nanoparticles can emulate essential biological functions, drawing from an abundant reservoir of cellular capabilities. This includes engaging in biological binding, precise homing to tumor sites, and interaction with immune cells. These inherent traits endow biomimetic nanoparticles with a suite of intelligent features, including biocompatibility, low immunogenicity, reduced toxicity, immune evasion, prolonged circulation, homotypic binding, enhanced tumor targeting, and the capability of precise delivery. By integrating biologically inspired coatings derived from cell membranes with nanoparticle cores, these carriers become highly versatile vessels for encapsulating a wide array of therapeutic agents. As a result, they are being extensively harnessed for the precise delivery of drugs and genes, underpinning numerous biomedical applications. This discussion delves into the challenges and opportunities presented by biomimetic nanoparticles and offers a comprehensive exploration of their fundamentals and recent breakthroughs, with an eye towards clinical translation. By bridging the gap between scientific innovation and clinical utility, biomimetic nanoparticles hold great promise for advancing the field of cancer treatment.

## Introduction

Cancer is a complex disease, which involves numerous cells and their crosstalk with surrounding environment, including immunosuppression in T cells via PD-1/PD-L1 axis, recruitment of stem cells via CXCR4/CXCL2 chemokine axis, maturation of immune cells via membrane interactions, and various other physical/chemical interactions, uncover the emergence of cell membrane-based drug delivery systems [1–2]. Cancer treatment has been revolutionized, yet cancer is treated with traditional methods, that is, chemotherapy, radiotherapy, and surgical intervention, accompanied by several lethal implications along with low solubility, poor bioavailability, and fatal off-target effects [3–4]. In addition, the escalation of new glitches such as drug sensitivity in tumor cells has been reduced due to the emergence of multidrug resistance (MDR) by various factors, including ATP-dependent drug efflux, selective stress of drugs, altered DNA repair mechanisms, cellular heterogeneity, recurrence, and altered metabolic responses inevitably leads to treatment failure [5–7]. Anyhow, an early detection of cancer enhances treatment success and increases survival. However, monotherapies proved limited therapeutic efficacy. Thus, an effective multiple cancer therapeutic regimen has been employed to successfully eradicate tumors [8]. To overcome these discrepancies, an efficient, biocompatible, nontoxic, non-immunogenic and precisely targeted drug delivery system is desirable [9].

Conventional non-targeted delivery systems result in off-targeting as they also affect healthy cells and organs. Therefore, there is an ultimate need to produce suitable carriers, which can reduce the side effects and toxicity, while achieving high therapeutic efficacy. Consequently, the use of nanoparticles (NPs) has been proven a great breakthrough in the field of cancer treatment. NPs, smaller than 100 nm, show unique physicochemical and biological properties, and incredible potential of being therapeutic agent carriers for biomedical applications [10–11]. They are capable to deliver a range of therapeutics including genes, vaccines, biological macromolecules, hydrophobic/hydrophilic drugs, and proteins to certain organs such as brain, liver, spleen, lungs, arterial walls for both immediate and sustained release. Their degradation and release kinetics can be controlled or manipulated by different methods and incorporation or conjugation of specific materials. Importantly, they mainly focus on different biomaterials, drug release behavior, targeting ability, and surface modifications [12–15].

A variety of nanoparticles have been researched including liposomes, polymer NPs, solid lipid NPs, and hybrid NPs [16]. Nanoscale drug carriers with the advantage of high penetration, long circulation, and significant targetability have been employed for the treatment of various fatal diseases such as cancer, Alzheimer’s, stroke, and diabetes [17–19]. However, the development of optimum NP drug carriers is still critical as they all come with several limitations. For example, liposomes can carry hydrophilic drugs that quickly eliminate from the system. Also, metal or polymeric NPs face poor biodegradability, which enriches them in kidney and liver tissues for longer than required, causing toxic effects while showing inadequate efficacy at the desired site [20–21].

**Figure 1:**
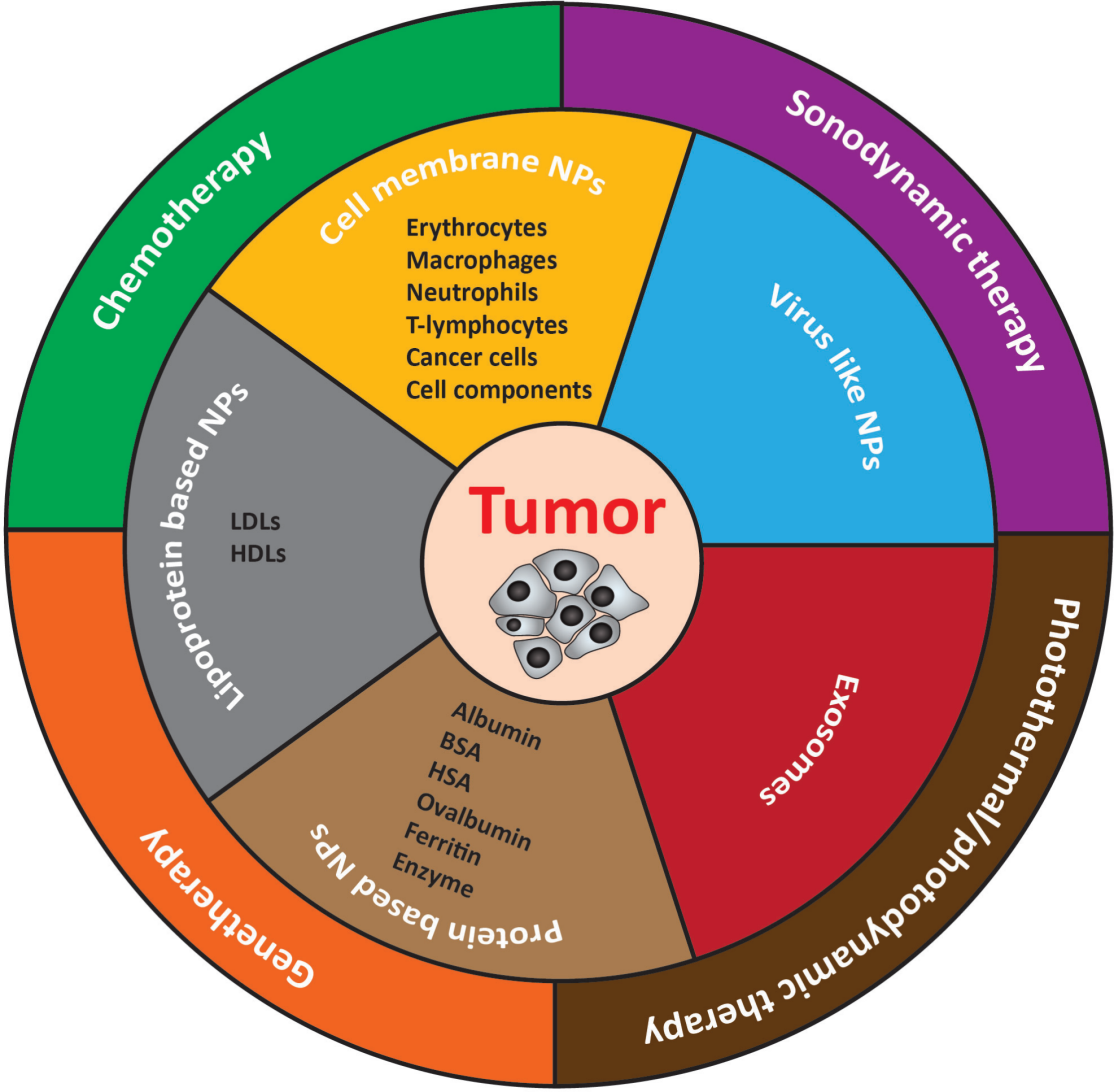
Types and antitumor application of biomimetic nanomedicines.

Biomimetic NPs potentially fit in this scenario. These are specialized NPs, where the surface is designed with natural biocompatible biomaterials that can mimic the structure and functions of the natural cells to improve targetability, enhance biocompatibility, and increase retention time with minimum undesired immune reaction [22]. Importantly, efficient tracking and profound interactions within complex biological environments can be achieved by using biomimetic NPs with prolonged circulation time, summarized in Figure 1.

**Figure 2:**
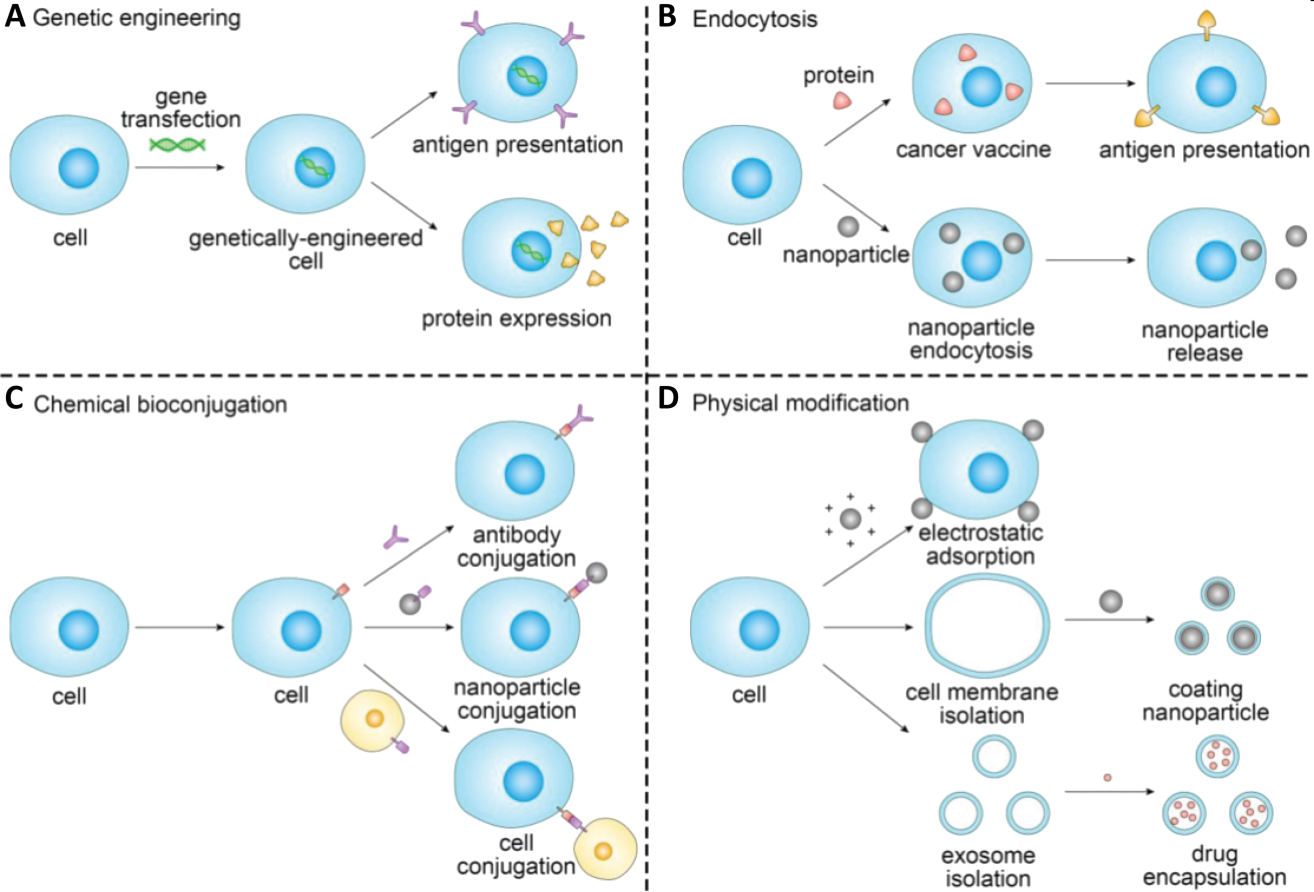
Schematic of the representative strategies of engineering cells for cancer immunotherapy. The cells used for drug delivery and cancer immunotherapy involve erythrocytes, platelets, leukocytes, cancer cells and stem cells. Figure 2 was reproduced from [23] (© 2019 X. Xu et al., published by Ivyspring International Publisher, distributed under the terms of the Creative Commons Attribution 4.0 International License, https://creativecommons.org/licenses/by/4.0).

Moreover, research in NP-mediated drug delivery has been shifted to focus on the use of cell-derived cancer therapies/engineered or live cells for cancer therapies and immunomodulation. In terms of drug discovery and delivery, various strategies have been developed including cell-based drug delivery, which provides a promising platform to enhance therapeutic drug delivery, increase efficacy, reduce off-targeting and side effects of the therapeutic regimen. By utilizing recent advances in micro/nanotechnology and molecular pharmaceutics, potent bioinspired cell-based therapeutics could be obtained by genetic engineering, endocytosis, chemical bioconjugation, and physical modifications (Figure 2) [23]. More precisely, cell membranes or whole cells, which could exploit homotypic targeting mechanism and exosomes, are being employed to deliver anticancer/immune modulating drugs or vaccine delivery [2,24]. The cell or biomimetic NPs can alter biological functions via various pathways and can be effectively used in targeting and manipulating their action site to achieve desired therapeutic effects. Thus, it is critical to explore and understand the types, targeting mechanism, immune responses, and therapeutic outcomes of such NPs. Unlike previous reports, here, we provide an up-to-date review of recent advances in biomimetic nanomedicines with a specific focus on their types and recent advancements only for cancer treatment.

This review focuses on the recent advancements in biomimetic nanomedicines engineered with various biomaterials, emphasizing their interactions with different types of tumors and tumor microenvironment (TME). It presents the role of biomimetic nanoparticles in developing targeted cancer therapies by selectively eliminating the tumor cells, sparing healthy tissues, and possibly stimulating the immune system. The review explores biomimetic nanodrug delivery systems as antitumor immunotherapy, including antigen/adjuvant delivery and tumor antigen-specific T-cell targeting strategies. It also summarizes the characteristics of biomimetic drug delivery nanocarriers designed from different cell types, their modification with specific ligands for precise and enhanced tumor targeting and TME responsiveness. Finally, by integrating nature-inspired architectures, these smart, multifunctional biomimetic nanoparticles offer a promising path to overcome current therapeutic challenges and revolutionize precision oncology.

## Review

### 1 Types of biomimetic nanoparticles

#### 1.1 Cell membrane-camouflaged nanoparticles

Cell membranes have emerged as an ideal strategy to protect synthetic nanoparticles during circulation. Cell membrane-coated biomimetic nanoparticles act like source cells with significant biomedical properties including biocompatibility, low toxicity, and potent targetability. They are recognized as “own” by the immune system and are not phagocytosed, thus increasing circulation and retention time [25].

**Figure 3:**
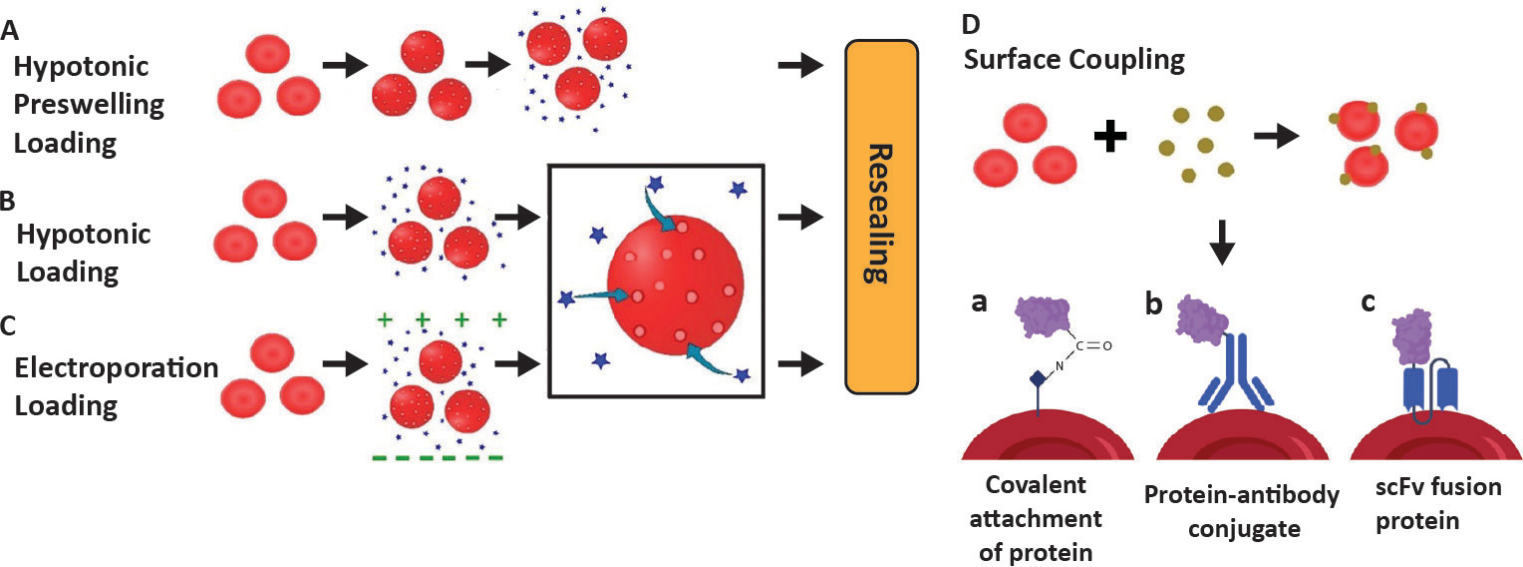
Drug loading strategies in erythrocytes. (A) Preswelling in hypotonic solution containing an excess of the drug, (B) drug loading in hypotonic solution containing an excess of the drug, (C) electroporation-mediated drug loading in isotonic solution containing an excess of the drug, and (D) hitchhiking drug or drug carrier attached on the surface of erythrocyte. Panels a–c represent three different methods of RBCs coupling with therapeutic proteins. Figure 3 D was reproduced from [30] (© 2021 J. S. Brenner et al., published by Annual Reviews, distributed under the terms of the Creative Commons Attribution 4.0 International License, https://creativecommons.org/licenses/by/4.0).

**1.1.1 Erythrocytes.** Erythrocytes (red blood cells, RBCs) are the most abundant form of cells in the blood and an excellent candidate for long-time circulation of drug carriers. Among all eukaryotic cells, they are easiest to use for biomedical applications as they lack mature DNA and other organelles [26]. There are various methods to load agents inside or attach onto the surface of RBCs by either chemical or physical methods such as (A) hypotonic presealing, (B) hypotonic loading, (C) electroporation, and (D) surface coupling (Figure 3). Generally, RBC membranes can be separated by hypotonic treatment to remove intracellular components (Figure 3A,B). Erythrocyte drug loading has been demonstrated by encapsulating artemether as a model drug and using different modifications of the hypotonic methods [27]. When exposed to a hypotonic solution, water enters the erythrocytes, an equilibrium is established as substances enter and leave the swollen RBCs. A hypotonic solution is prepared by dissolving a relatively higher amount of the drug. Drug loading can be enhanced by carrying out the process at 0 °C as the fatty acid chains in the membrane solidify and pores remain open for a longer period. In contrast, storage of erythrocytes at 0 °C before drug loading prevents pore formation, leading to lowered drug loading. Finally, the tonicity of the solution is raised to attain isotonic conditions, and the rising temperature leads to the resealing of RBCs. It was also observed that optimum drug loading is achieved at a certain tonicity level. Therefore, artemether loading was higher when using 0.6% NaCl hypotonic solution than when using 0.3% NaCl solution [28]. Drugs can also be loaded in erythrocytes by electroporation (Figure 3C). Although electroporation is an easy and feasible loading method, its scale-up is challenging [29]. Many researchers have attached drugs, therapeutic proteins, or drug-loaded nanoparticles onto the surface of RBCs to transfer innate characteristics of RBCs to the nanoparticles (Figure 3D) [30].

**1.1.2 Macrophages.** Macrophages are a critical part of the TME. They are specific type of immune cells that can recognize healthy somatic cells as “own” cells and exogenous particles, cancer cells, and pathogens as “foreign” and phagocytose them [31]. Therefore, macrophage membranes could be used to escape the reticuloendothelial system (RES), target cancer cells and pathogens, and enhance tumor accumulation. Recently, Huang et al. reported macrophage membrane-coated targeted NPs for tumor inhibition and macrophage polarization. They incorporated methyltransferase like 14 (METTL14) and RS09 inside the macrophages and then functionalized the surface with DSPE-PEG2000-cRGD. METTL14 significantly inhibits the tumor growth in vitro, downregulates TICAM2, and inhibits the macrophage polarization by the Toll-like receptor 4 pathway. The combinatory NPs induce antitumor M1 macrophage polarization, and cRGD modification further enhances tumor accumulation [32].

**Figure 4:**
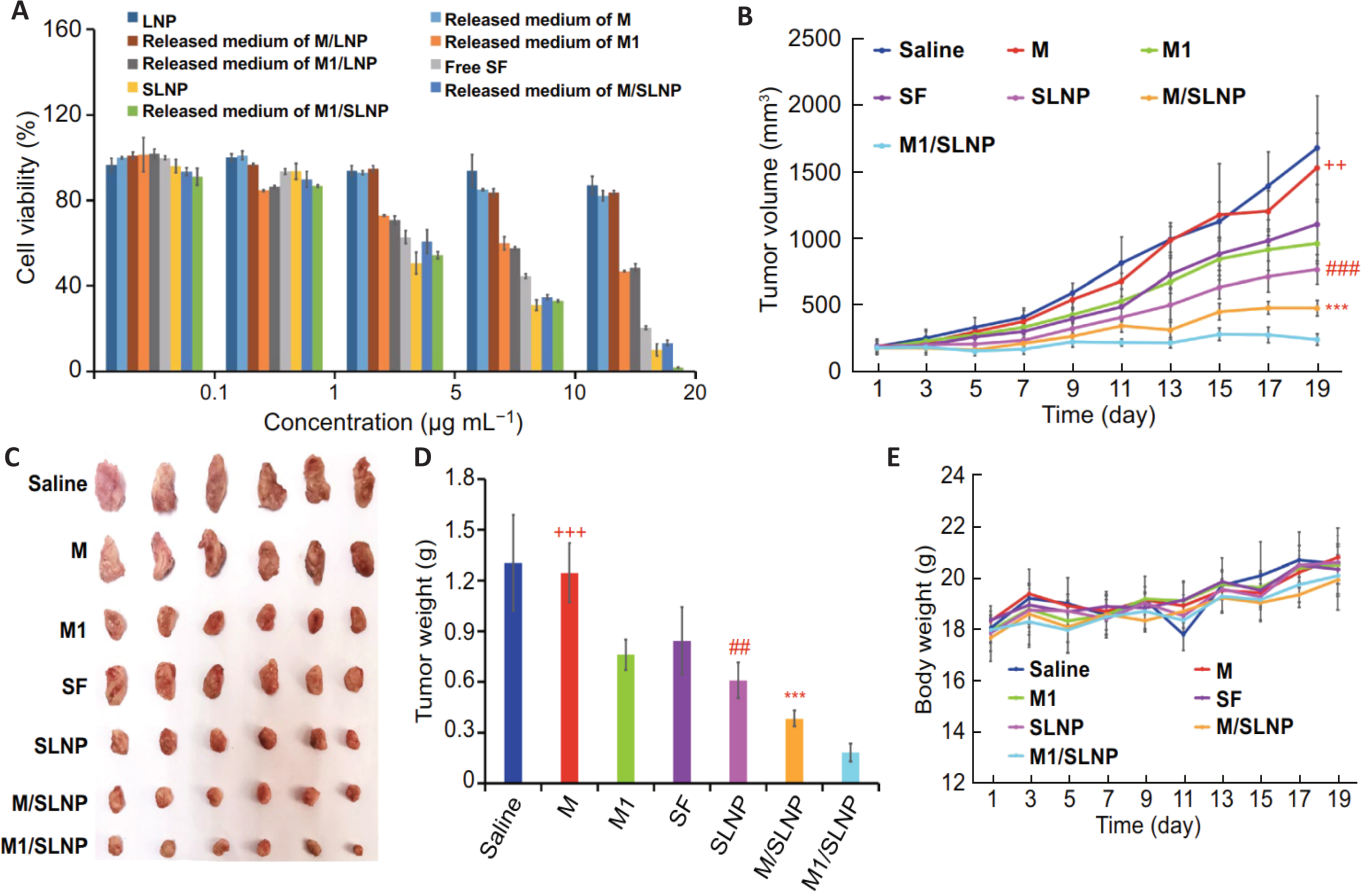
In vitro and in vivo antitumor efficacy of M1/SLNP. (A) Cell viability in Hepa1-6 cells, (B–D) changes in tumor volume and weights, and (E) body weight changes in Hepa1-6 tumor bearing mice. Figure 4 was reproduced from [33] (© 2021 T. Hou et al., published by Springer Nature, distributed under the terms of the Creative Commons Attribution 4.0 International License, https://creativecommons.org/licenses/by/4.0).

In another study, Hou et al. employed M1-type macrophages and loaded them with sorafenib (SF) to develop lipid nanoparticles (M1/SLNPs). The M1/SLNPs showed an increase in tumor accumulation and enhanced the SF tumor targeting efficacy. Furthermore, they increased the ratio of M1-type macrophages, CD3+CD4+T cells, and CD3+CD8+T cells in the tumor tissues, indicating the reversal of immunosuppressive TMEs (Figure 4) [33]. Interestingly, Xue et al. applied magnetothermal (MHT) antitumor therapy because of minimal invasiveness, high efficiency, and better tissue penetration. They developed small Fe@Fe_3_O_4_-DHCA nanoparticles (≈14 nm) and coated them with macrophages (RAW267.4 cells) for magnetic resonance imaging (MRI) and MHT of solid tumors. The Fe@Fe_3_O_4_-DHCA NPs showed accumulation in tumor cells resulting in enhanced MRI and MHT performance in vitro. Furthermore, RAW267.4-loaded with Fe@Fe_3_O_4_-DHCA demonstrated efficiency in vivo. Thus, Fe@Fe_3_O_4_-DHCA nanoparticles showed great application potential for tumor diagnosis and therapy [34].

**1.1.3 Neutrophils.** Neutrophils are among the most abundant and frontline phagocytes among the white blood cells [35]. Neutrophils are the first to appear at the site of inflammation and easily cross different biological barriers such as blood–tissue barrier, blood–brain barrier (BBB) or blood–tumor barrier (BTB). Therefore, neutrophil membrane-coated nanoparticles have been successfully employed to cross biological barriers to target cancer cells. The neutrophil membranes enhanced circulation life, efficiently targeted tumors, and inhibited growth by photodynamic therapy. Neutrophil membrane-coated celastrol-loaded PEG-PLGA nanoparticles were prepared to treat pancreatic cancer, where drug delivery is limited by the blood–pancreas barrier. The nanoparticles successfully crossed the barrier, accumulated selectively in pancreatic cancer cells, inhibited tumor growth and its metastasis to the liver [36]. Similarly, neutrophil membrane-coated PLGA nanoparticles were used for the combination of near-IR imaging and photodynamic therapy of hepatocellular carcinoma [37].

**1.1.4 T lymphocytes (T cells).** T cells are highly specific and more actively identify foreign bodies, including cancer cells. In addition to directly killing cancer cells, T cells organize the immune response by recruiting other immune cells and also prevent the immune response from overreacting [38]. Therefore, T cells are the most extensively studied immune cells in cancer immunotherapy [39]. Kang et al. have developed T lymphocyte membrane-coated nanoparticles that can target cancer by T cell-associated proteins and kill cancer by cytotoxic agents loaded in the nanoparticles. Unlike T cells, the T lymphocyte membrane-coated nanoparticles are resistant to immunosuppressive and apoptotic signaling [40]. In recent years, the applications of T cell-mediated cancer cell targeting have expanded. One example is intravenous administration of T cell membrane-coated nanoparticles directed to the cancerous organ by an externally applied magnetic field, followed by immune cell membrane-mediated cancer targeting. This strategy led to accelerated accumulation of nanomedicine in the tumor with minimal off-target exposure [41]. Wayteck et al. have prepared liposomes that can hitchhike on cells to the tumor site and get separated to perform their cytotoxic activity [42].

**1.1.5 Cancer cells.** Cancer cells establish their own mechanism to escape immune response [43]. They are tightly bound by surface proteins to hinder the penetration of drugs and drug carriers. In addition, cancer cell membranes express specific receptors and antigens that help them recognize and target through a homotypic binding mechanism [44–45]. Therefore, cancer cell membranes are also extensively explored to localize nanomedicines to cancer cells. Cancer cell membrane-coated nanoparticles can enter the cancer cells simply by fusion. The membrane coating is fused with the cancer cell membrane and delivers the payload inside the cytosol [46]. A distinctive characteristic of cell membrane-coated nanocarriers is that they include carbohydrates, proteins, and lipids, in addition to being biocompatible [47]. For this reason, cancer membranes are also preferred for vaccine delivery [48]. Some researchers incorporated RBC membrane components into cancer cell membranes to form a hybrid membrane, which facilitates simultaneously circulation in blood after intravenous administration and fusion with cancer cells to deliver payload intracellularly (hybrid cell membrane). However, the cell membrane-coated nanoparticles targeting efficiency is not universal as it does not always result in successful targeting, may be due to host–donor mismatch or intra-patient differences in cancer cell expression. This problem could be solved by using patient-derived cancer membranes for personalized cancer treatment [49].

**1.1.6 Cell component platelets.** Platelets lack a nucleus and are the smallest of all blood components. They circulate freely in the body and can reach deep tissues. Importantly, platelets are reported to activate and specifically bind to cancer cells [50]. Therefore, platelets have been used to load cytotoxic drugs for prolonged circulation in blood, escape immune response, and reach the tumor site [51]. Due to their small size, platelet-based NPs can be made as small as 175 nm [52]. Notably, they can be stored at −80 °C while preserving their cytotoxic and targeting functions [53]. Tang et al. have prepared Janus platelet microrobots that use asymmetrically surface-attached urea enzyme to drive them through the body fluids. The asymmetrical catalysis of urea to ammonia acts as a gas jet to propel the microrobots to the desired site of action [54].

#### 1.2 Lipoprotein-based biomimetic nanoparticles

Lipoproteins are complex structures with a lipid core, usually cholesterol esters and triglycerides, surrounded by a monolayer of phospholipids and apolipoproteins [55]. Lipoproteins include, among others, low-density lipoprotein (LDL) and high-density lipoprotein (HDL). Due to the hydrophobic core and prolonged circulation, they have been used in drug delivery. The lipid core of the lipoprotein tend to load a variety of lipophilic drug molecules [56–57], while the apolipoproteins guide the formation of lipoproteins, provide structural integrity, activate or inhibit lipoprotein metabolism, and act as ligands for lipoprotein receptors. Moreover, they play an important role in tumor regulation and progression, as they are potent and specific inhibitors of 3-hydroxy-3-methylglutaryl coenzyme A reductase, the rate-limiting enzyme of the mevalonate metabolic pathway [58]. The use of statins, drugs that lower plasma lipoprotein levels, has been shown to reduce the risk of certain types of cancer [59–60].

**1.2.1 Low-density lipoprotein (LDL).** Lipoproteins can load small molecular drugs (including chemotherapeutic agents), nucleic acids, and other macromolecules [61]. LDL was the first lipoprotein used for drug delivery and imaging applications. The particles are smaller than 50 nm and are characterized by surface apolipoprotein B-100. The uptake of LDL inside the cells occurs mainly via receptor-mediated endocytosis by a structurally similar receptor family, similar to LDL receptor proteins including LDL receptor-related protein (LRP or megalin), very-low density lipoprotein (vLDL) receptor, and apolipoprotein E receptor-2 (ApoER2) [62]. After uptake, LDL is phagocytosed to form lysosomes. However, lysosomes can degrade most drugs, which limits their applications [63–64]. Zhu et al. proposed reconstituted LDL nanoparticles to prevent acidic degradation. They added fatty acids to encapsulate drugs in the lipid core because fatty acids are not degraded in the lysosome and can safeguard the payload [64].

**1.2.2 High-density lipoprotein (HDL).** HDL plays an important role in cancer regulation due to its effect on immunomodulation, and anti-inflammatory and antioxidant action to suppress tumor growth [65]. Additionally, HDL can modulate the tumor microenvironment and reverse cholesterol transportation to cancer cells to limit their growth [66]. Consequently, HDL has been reported as a therapeutic agent to alleviate certain types of cancers. Recently, Rink et al. have prepared HDL nanoparticles that target SCARB1, inhibit cholesterol uptake, and induce ferroptosis of the cancer cells [67]. HDL presents many features that make it ideal for drug delivery applications including biocompatibility and biodegradability, long circulation, hydrophobic core, and small size. The main lipoprotein of HDL is alpha apolipoprotein (apo A-I and apo A-II) that can bind SR-BI receptors overexpressed on cancer cells. Unlike LDL, HDL is transported directly into the cytosol bypassing lysosomal degradation [68]. Reconstituted HDL (rHDL) is now developed by changing the chemical composition or morphology to optimize drug delivery. When loaded with chemotherapeutic agents, rHDL shows outstanding active targeting and anticancer activities [57]. Moreover, rHDL showed potential to co-load hydrophobic paclitaxel and hydrophilic doxorubicin in apo A-I targeted rHDL nanoparticles. These nanoparticles showed superior antitumor activity in vitro and in vivo [69]. Previously, rHDL combined with a hydrophilic polymeric core and a magnetic core for imaging applications [70–71].

**Figure 5:**
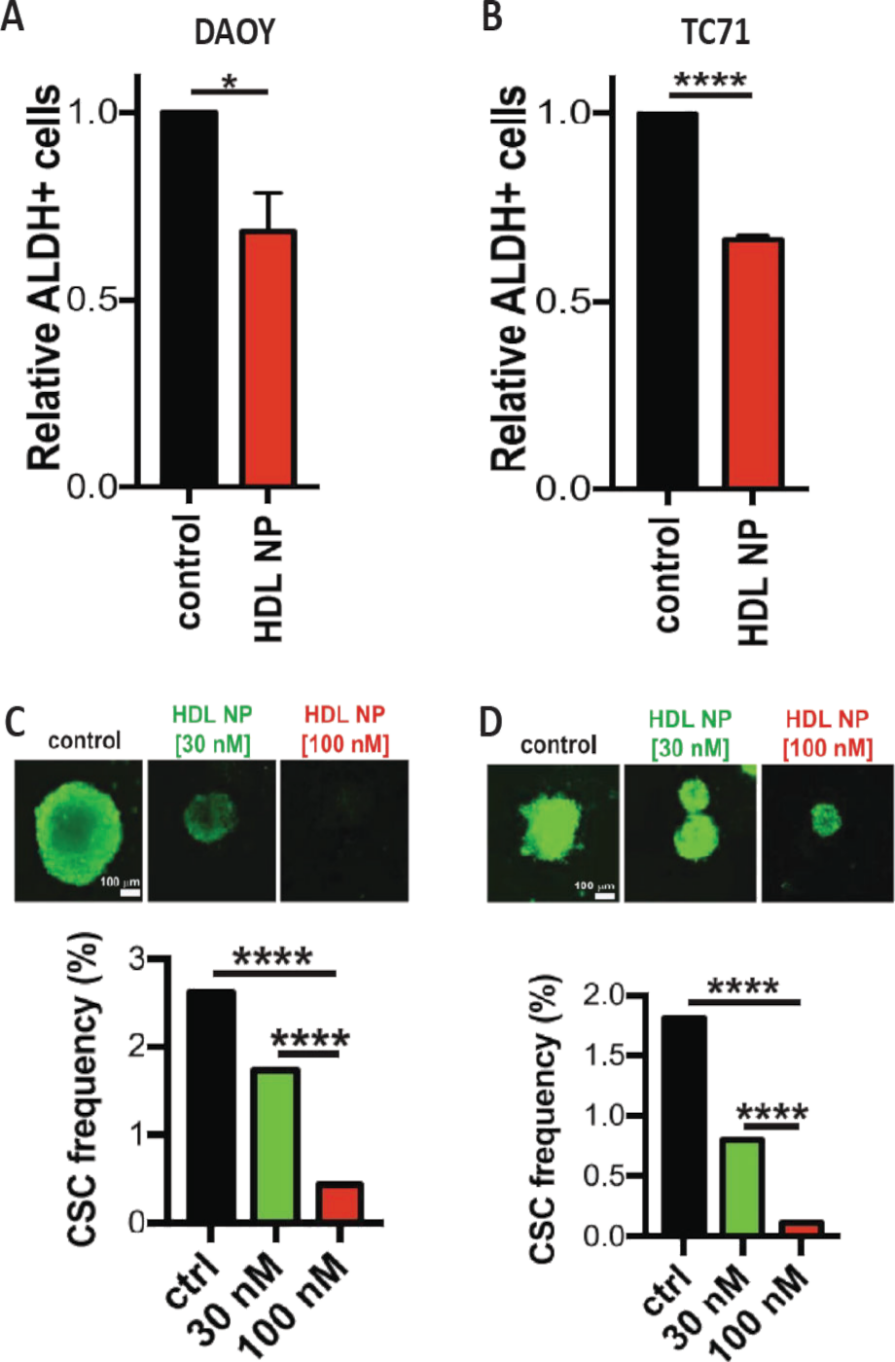
Cancer stem cell depletion with HDL NPs. (A, B) DAOY and TC71 cells were treated with or without HDL NPs depicting the proportion of ALDH+ cells relative to control, (C, D) DAOY or TC71 cells seeded in the presence or absence of HDL NPs to assess sphere formation. Figure 5 was adapted from [72] (© 2018 J. B. Bell et al., published by Springer Nature, distributed under the terms of the Creative Commons Attribution 4.0 International License, https://creativecommons.org/licenses/by/4.0).

Cholesterol has been associated as a key player in the emergence of many diseases including medulloblastoma. HDL nanoparticles have been prepared to treat medulloblastoma by disrupting the cholesterol signaling pathway (Figure 5). The HDL nanoparticles were successfully taken up by the medulloblastoma cells (DAOY) and hedgehog-driven Ewing sarcoma cells. Results showed that HDL nanoparticles induce efflux of cholesterol to lower its intracellular level and induce tumor cell death by disrupting cholesterol signaling [72]. Therefore, HDL nanoparticles not only an effective drug carrier with inherent targeting ability but can also act as a therapeutic agent against cholesterol-dependent diseases.

#### 1.3 Protein-based biomimetic nanoparticles

Peptides and proteins are essential to maintain hemostasis by binding various biomolecules circulating in blood. They not only maintain the electrolyte and osmotic pressure but also deliver a variety of molecules across the body [73–74]. Peptides possess different functional groups on their surface that can act as a template for various NPs, mostly for diagnostic or multifunctional theranostic applications.

**1.3.1 Albumin.** Albumin is a major protein present in blood and widely studied for drug–protein interaction and nanoparticle corona formation studies. Due to its immunocompatibility, long half-life, and abundance of binding sites it is considered safe for drug delivery applications. In fact, albumin-conjugated drugs are already used in clinical practice [75]. Albumin molecules can be used as a template to surface-decorate inorganic/metallic nanoparticles by biomineralization. In this method, the thiol group at the albumin surface acts as scaffold for synthesis of nanoparticles [76]. The thiol groups are protonated at neutral pH and can entrap Au(III) ions. When the pH is changed to basic (≈12), Au(III) is reduced to Au and gold nanoparticles are formed [77]. Synthesis of albumin-templated nanomaterials depends upon many factors. Yang et al. produced ceria nanoparticles, nanoclusters, and nanochains by changing molar concentration, time of reaction, and temperature. They found that size and morphology of the nanomaterials can be optimized by careful tuning of switchable ionic redox systems (Ce^3+^/Ce^4+^), the unique structure of protein, and reducible disulfide groups [78–80].

**1.3.2 Bovine serum albumin.** Bovine serum albumin (BSA) is widely used in biomedical applications such as supplemental growth media and protein standards. BSA was used as a template for the synthesis of organic–inorganic hybrid nanoparticles. BSA has some immunogenic effects and is therefore used in immunotherapy and analytic bioassay applications [78,81–83].

**1.3.3 Human serum albumin.** Human serum albumin (HSA) has biomedical applications as HAS-templated NPs exhibit immune escape, enhanced stability, and high drug loading [84–87]. HSA NPs can load drugs and contrast agents directly and prolong their circulation [88]. Furthermore, cancer cells overexpress albumin receptors on their surface. Therefore, albumin nanoparticles have an intrinsic ability to target cells by fusion to the surface proteins [89].

Just like drug–protein binding in vivo, nanoparticles can adsorb plasma proteins at their surface in blood circulation and form a corona, which can alter their biodistribution, cell uptake, and intracellular degradation [90]. Thus, as the protein corona increases, albumin proteins affect nanoparticle fate in vivo. As albumin is the most abundant protein in the blood, precoating with albumin has been shown to prevent adsorption of other plasma proteins and degradation of nanoparticles in vivo [91–92].

**1.3.4 Ovalbumin.** Ovalbumin (OVA) is a major component of egg white and a readily available protein for drug delivery applications. It forms crosslinked gels and responds to changes in pH and temperature [93]. To escape the RES, ovalbumin nanoparticles are conjugated with polyethylene glycol. Controlling the PEG/OVA ratio allows for fine-tuning of critical physical properties, such as particle size, elasticity, and mesh size [94]. Like BSA, ovalbumin can activate an immune response and the presence of endotoxin contaminants activates macrophages and dendritic cells [95]. Therefore, ovalbumin nanoparticles are generally limited to immunotherapeutic applications.

**1.3.5 Ferritin-based biomimetic nanoparticles.** Ferritin is an iron transport protein and possesses a hollow cage-like structure, which offers high drug loading. Initially, ferritin was only used as a template to develop diagnostic agents [96]. Later, it was used to encapsulate a wide range of therapeutic agents. Importantly, ferritin receptors are overexpressed in cancer cells and serve as a platform for active targeting by using ferritin nanoparticles. Bellini et al. synthesized ferritin nanoparticles for in vitro and in vivo targeting of cancer cells [97]. As ferritin is prepared by genetic engineering, it can be genetically modified to recombinant ferritin. This property was exploited to design redox and pH dual-responsive ferritin nanoparticles. In drug-loaded mesoporous silica nanoparticles, ferritin was used as a gating material. It covered the pores to prevent drug release and opened only when activated by redox or pH stimuli [98]. Similarly, recombinant ferritin can be used as a carrier of macromolecules such as enzymes by electrostatic interaction with the negatively charged interior of the ferritin cage [99].

**1.3.6 Enzyme-based biomimetic nanoparticles.** Enzymes are biological catalysts and proteins by nature. Multienzymes are complex structures in which enzymes that perform sequential functions of a metabolic pathway are noncovalently associated with each other. The multifunctional enzyme complexes inspired researchers to design multienzyme complexes of their own choice. Initially, multiple enzymes were co-loaded in liposomes to perform different steps of a metabolic pathway [100]. Later, this strategy has evolved into the development of artificial organelles, which contain enzymes for a complex cascade reaction. Artificial organelles consist of capsosomes with a polymeric shell containing multiple liposomes, where each liposome may carry a certain type of enzyme. The capsosomes can efficiently enter the cell, and the enzymes exert their specific activities intracellularly [101]. More recently, the microfluidic spray technique was used to load alcohol oxidase and catalase into hollow hydrogel microspheres. The microfluidic spray technique provided excellent control over the number, type, and spatial control of the enzyme. The enzyme cascade was successfully applied to reduce the alcohol levels as an alternative antidot for alcohol intoxication [102].

#### 1.4 Exosome-based biomimetic nanoparticles

Exosomes are endogenous extracellular vehicles released by most of the cells. These phospholipid bilayer nanovesicles are surface-enriched with proteins accounting for their dynamic and prominent roles in immune escape, cell–cell communication, and specific cell uptake [103]. They are small in size (30–150 nm), bypass biological barriers, and are efficiently taken up by cells due to their surface markers [104]. The International Society for Extracellular Vesicles has set out guidelines on the purity of extracellular vesicles depending upon intended applications [105]. Through exosomes, donor cells can transfer exogenous substances, such as proteins, mRNAs, microRNAs (miRNAs), and lipids, to recipient cells. Consequently, these naturally equipped nanocarriers have been used for drug delivery [106].

Exosomes are usually isolated from biological samples by centrifugation, size exclusion chromatography, ultrafiltration, immune affinity, and commercial kits [107]. Microfluidic devices are now aiding the separation of exosomes by acoustic cell separation [108–109]. The drugs can be either directly load in exosomes or exosomes are sliced to remove contents and reassembled [110]. The interaction between exosomes and host cells is very complex involving recognition, binding, cellular uptake, and intracellular transport. These steps are specific to different types of target cells and determine the fate of exosomes [107]. Moreover, they offer prolonged circulation, excellent target specificity, and intracellular delivery without degradation. Exosomes have also been used to deliver chemotherapeutic agents, nucleic acids, macromolecules, and diagnostic agents. Thus, exosomes are ideal delivery vehicles for therapeutic treatments that should be specific to the targeting sites with low toxicity to other organs, high encapsulation and delivery efficiencies. They protect the payload while in circulation and maintain a steady release profile [111].

#### 1.5 Virus-like particles

**Figure 6:**
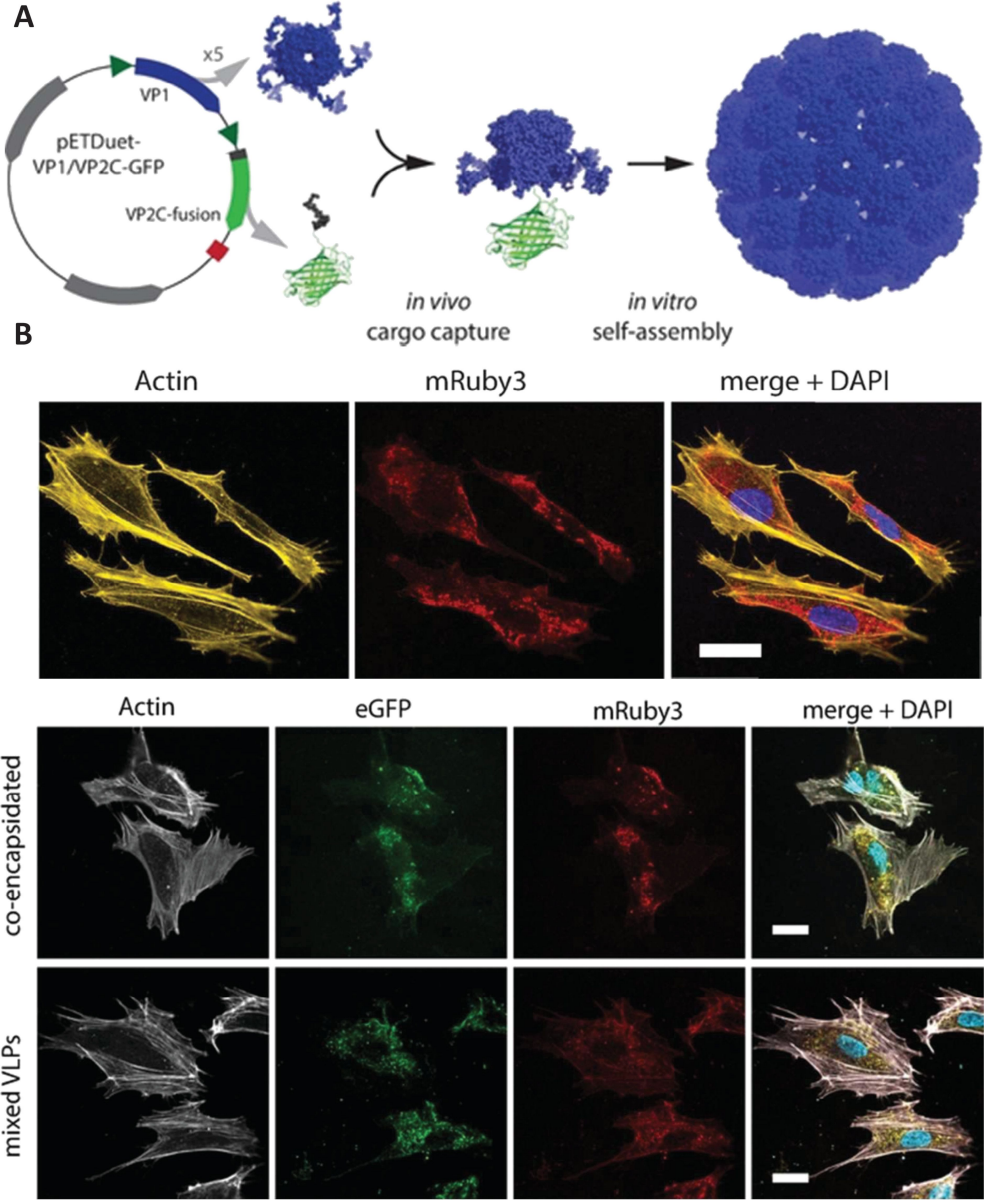
Schematic presentation of (A) cargo capture and self-assembly of virus-like particles, (B) cellular uptake of mRuby3-loaded VLPs, and (C) co-delivery of cargo proteins to HUVECs cells incubated with VLPs co-encapsulating GFP and mRuby3, or with a mixture of VLPs separately encapsulating the fluorescent proteins. Figure 6 was reprinted with permission from [114], Copyright 2018 American Chemical Society. This content is not subject to CC BY 4.0.

Virus or virus subunits have long been used as vaccines for the protection against various diseases. Nanotechnology has advanced this field by facilitating the safe delivery of vaccines and improving their immunogenicity [112]. The presence of viral nucleic acids has been a key concern in medical applications, which limits the use of viruses in living organisms. Virus-like particles are multimeric nanoparticles consisting of viral proteins but lack viral genetic material. Thus, they lack pathogenicity but offer virus-associated specific tissue targeting and intracellular delivery of payload [113]. For example, Dashti et al. reported a novel method to encapsulate guest proteins in murine polyoma virus capsomeres; the virus capsomere was prepared by expression of viral protein in recombinant *Escherichia coli*. Then the virus capsomere was transferred to a dialysis bag and allowed to self-assemble into nanoparticles in assembly buffer. The virus-like particles efficiently protected the payload and successfully delivered multiple proteins directly into cytosol of the cells [114] and induced a strong immune response due to the presence of viral epitopes. Plant virus-like particles have been used to treat canine oral melanoma using radio-immunotherapy (Figure 6).

Bacteriophages are also widely used for drug delivery applications. M13 phage is the most extensively studied phage for targeted drug delivery. In a recent study, the DNA of the M13 phage was modified to encode for SPARC binding peptide and cathepsin B cleavage peptide. Then, superparamagnetic iron oxide nanoparticles were covalently bonded to cathepsin B expressed on M13 phages to track their intracellular delivery [115]. Excellent control of shape, size, and genetic modification makes virus-like particles an excellent opportunity for safe and effective delivery of payload to an intended site.

### 2 Biomedical applications of biomimetic nanoparticles

#### 2.1 Chemotherapy

In recent years, the development of novel chemotherapeutics and their delivery vehicles has received great attention. The delivery strategies can significantly improve chemotherapeutics efficacy and significantly overcome poor bioavailability, adverse effects, and resistance. Nevertheless, drug delivery is still facing many challenges, including non-specific targeting, rapid clearance, limited accumulation at tumor sites, and a set of biological barriers that need to be passed. So far, scientists have put great effort into fabricating smart nanomaterials that could achieve targeted drug delivery [116–119]. In biological systems, drug delivery systems interact with different body structures as well as physiologic environments. Thus, the structures of drug carriers must be constructed in a way that allows them to elude immune recognition and overcome barriers via effective bio-interfacing.

**Figure 7:**
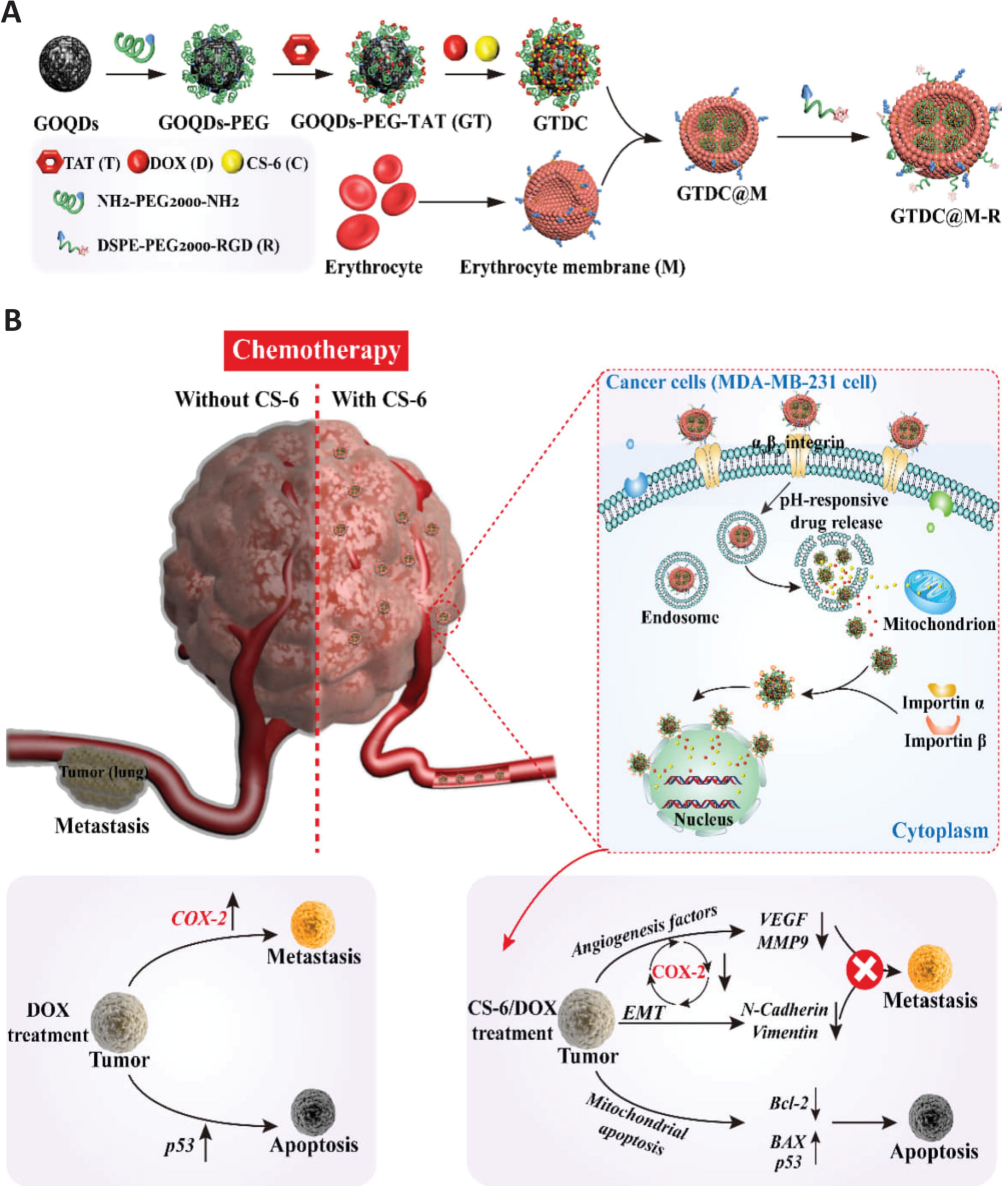
(A) Preparation of TAT and RGD modified RBC-covered graphene oxide quantum dot nanoparticles and (B) inhibition of tumor growth and metastasis mediated by the nanosystem and its proposed molecular mechanism. Figure 7 was reprinted from [128], *Acta Biomaterialia*, vol. 113, by J. Fan; B. Liu; Y. Long; Z. Wang; C. Tong; W. Wang; P. You; X. Liu, “Sequentially-targeted biomimetic nano drug system for triple-negative breast cancer ablation and lung metastasis inhibition”, Pages 554–569, Copyright (2020), with permission from Elsevier. This content is not subject to CC BY 4.0.

Within the scope of biomimetic nanomedicine for chemotherapeutic delivery, RBC-mimicking nanoparticles have been investigated extensively, as they are predominant cells in circulation, RBC membrane isolation is relatively easy, and they express various surface receptors that help immunocompatibility, immune evasion, and long half-life. RBC-mimicking nanostructures were investigated and successfully used for different tumor targeting and treatment applications [120–123]. In this respect, drug nanocrystals coated with RBC membrane and modified with a tumor-targeting peptide was successfully used for targeted therapy of glioma [124]. The peptide-modified nanosystem showed increased drug accumulation and enhanced therapeutic activity both in subcutaneous and orthotopic tumor models [125–126]. Another study regarding targeted therapy and improved drug delivery to the brain used the dual modification of RBC-coated lipid nanoparticles with T7 peptide, a ligand of transferrin receptor, and NGR peptide, a ligand of CD13 [127]. Dual modification with the peptides yielded the ability to overcome the BBB and target the glioma. In another study, RBC-covered graphene oxide quantum dots (GTDC@M) were investigated regarding the targeted therapy of metastatic breast cancer (Figure 7) [128]. The nanosystem was modified with TAT (T) and RGD (R) peptides for targeted delivery of the chemotherapeutics gamabufotalin (C) and doxorubicin (DOX) in triple-negative breast cancer (TNBC), resulting in potent inhibition of tumor growth and breast cancer metastasis. RBC membrane was also conjugated with gold nanostars to control the release with near-infrared irradiation. Also, RBC and platelet hybrid membrane-coated gold nanostars achieved targeted delivery to melanoma cells while avoiding macrophage phagocytosis [129].

**Figure 8:**
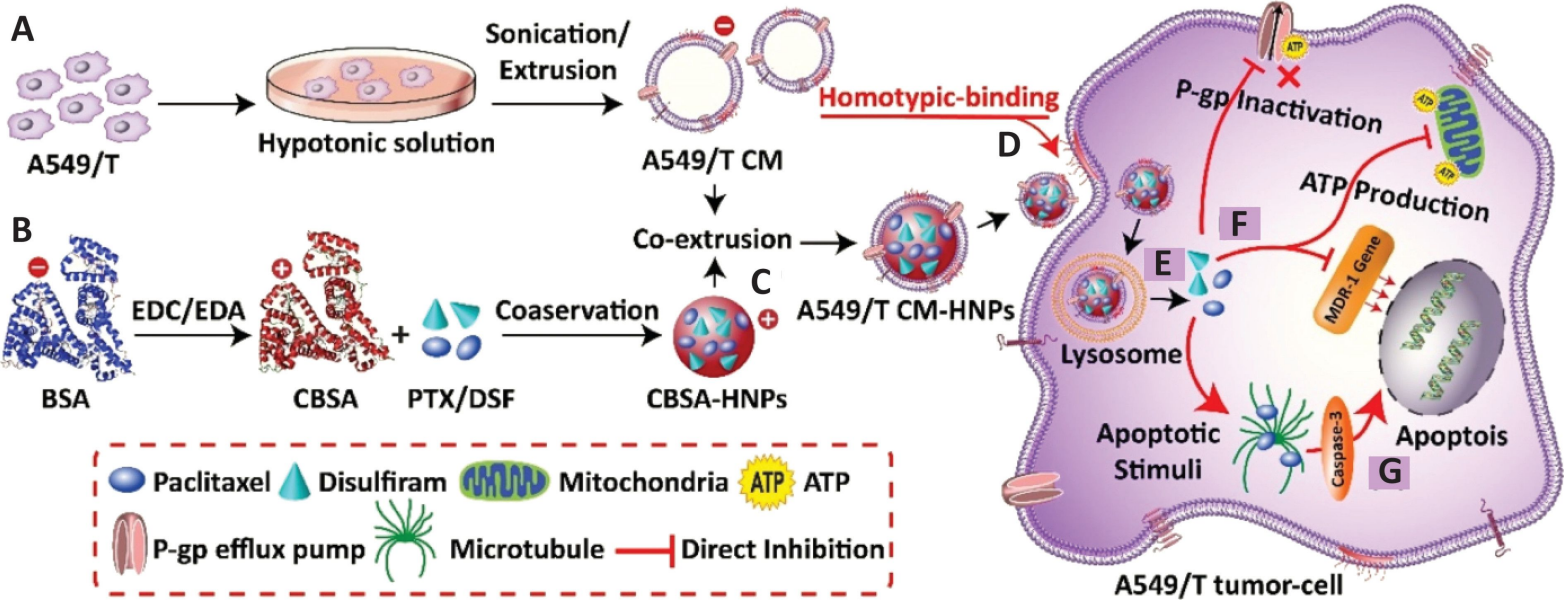
(A) Hypotonic treatment of taxol resistant A549/T cells to obtain A549/T cell membrane vesicles, (B) synthesis of cationic bovine serum albumin (CBSA), (C) hybrid nanoparticles (CBSA-HNPs) generation by sonication and co-extrusion, (D) A549/T CM-HNPs targeting via homotypic-binding mechanism, releasing (E) paclitaxel and disulﬁram, and (F) inhibited ATP concentration, downregulated MDR-1 gene, and therefore suppressed P-gp activity and increased intracellular PTX to induce (G) caspase-3-dependent MDR tumor cell apoptosis. Figure 8 was reproduced from [45] (©2020 Mohammad et al., published by MDPI, distributed under the terms of the Creative Commons Attribution 4.0 International License, https://creativecommons.org/licenses/by/4.0).

Although RBC-camouflage nanomedicines are feasible biomimetic nanoplatforms, other cell membrane-coated drug delivery systems also provide promising therapeutic strategies [130–132]. Nanomaterials camouflaged with cancer cell membrane (CCM) have been used for a number of theranostic applications [133–134]. Rao et al. showed that CCM-coated nanoparticles displayed excellent targeting ability when the nanoparticle-coating membrane matched with the tumor (host) [49]. In this study, a CCM coating of gelatin nanoparticles was employed against head and neck squamous cell carcinoma. The nanoparticles coated with patient-derived CCM provide effective targeting of the tumor in patient-derived tumor cells animal models through homologous targeting. In another study, the homologous targeting effect was successfully utilized to deliver doxorubicin (DOX), mefuparib hydrochloride (MHP) and poly(ADP-ribose) polymerase inhibitor in MCF7-tumor bearing mice [46]. In that study, a PEGylated-lipid nanoparticle (PEG-liposome) with a mesoporous silica nanoparticle core (LM) was prepared, and then the nanosystem was coated with CCM (CCM@LM). The biomimetic nanomedicine showed high internalization in a way similar to an enveloped virus. The PEGylation of the inner cavity provided subcellular localization of payload in the nucleus subsequent to cellular internalization. The whole nanosystem demonstrated a significant anti-tumor activity. Shao et al. established X-ray-responsive CCM-covered mesoporous organosilica nanoparticles for the controlled release of DOX [135]. The drug-loaded nanoparticle was camouflaged with 4T1 membrane, and the biomimetic system showed prolonged circulation, enhanced tumor accumulation, and release of DOX under low-dose radiation. Furthermore, Mohammad at al. exploited homotypic mechanisms to enhance the therapeutic efficacy of chemotherapeutics in lung cancer MDR cell lines. They isolated diﬀerent cell membranes (RBCs, 4T1, LO2, and A549-T) and constructed cell membrane-camouflaged biogenic nanoparticles to deliver antitumor paclitaxel and MDR-modulator disulﬁram. Consequently, the MDR cancer cell membrane-coated nanoparticles (A549/T CM-HNPs) selectively recognized the source cells and increased the cellular internalization up to nine-fold via homotypic binding. Moreover, the A549/T CM-HNPs sensitized MDR cells to PTX by suppressing the P-gp activity 3.2-fold and induced apoptosis (70%) in homologous A549/T cells [45] (Figure 8).

**Figure 9:**
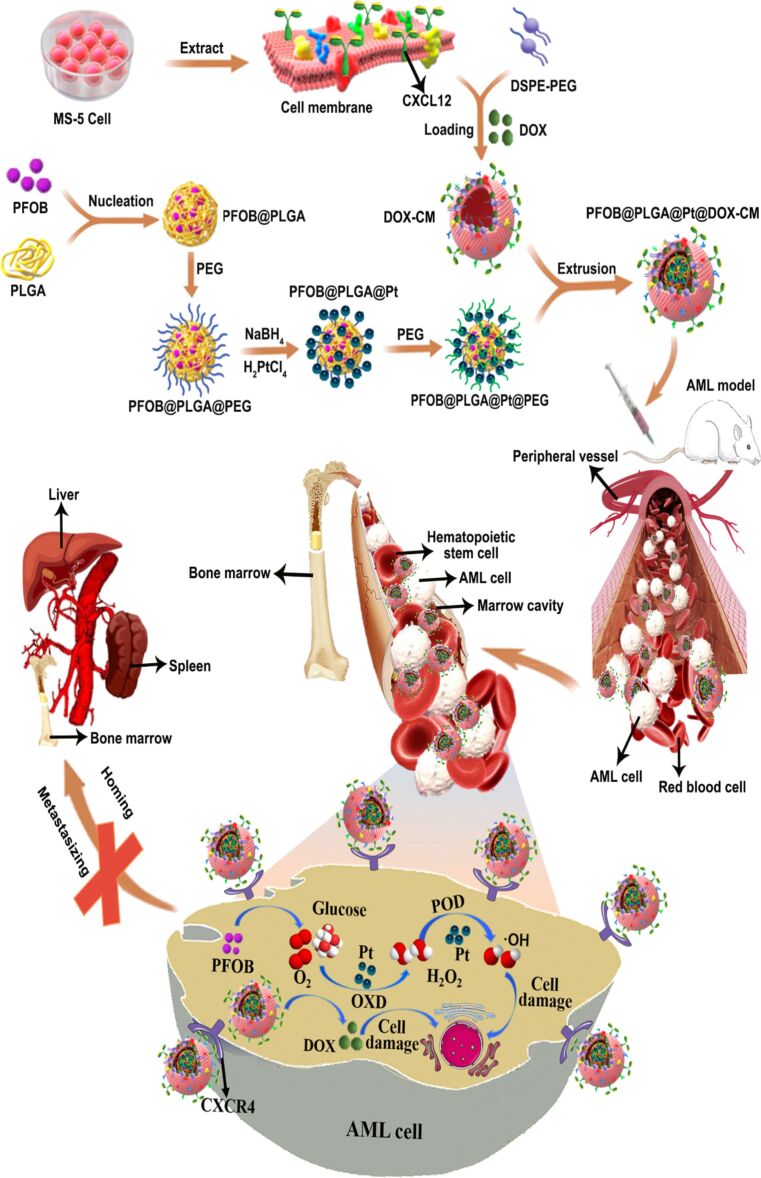
Schematic representation of biomimetic PFOB@PLGA@Pt@DOX-CM for anti-leukemia efficacy via chemotoxicity and excessive ROS generation to induce leukemia cell apoptosis. Furthermore, PFOB@PLGA@Pt@DOX-CM accumulate in the bone marrow and act as CXCR4 antagonists to block the leukemia cell-stroma adhesive interactions and prevent infiltration of AML cells to other normal organs, such as liver and spleen. Figure 9 was reproduced from [136] (© 2022 F. Kong et al., published by Elsevier B.V. on behalf of KeAi Communications Co. Ltd., distributed under the terms of the Creative Commons Attribution-NonCommercial-NoDerivatives 4.0 International License, https://creativecommons.org/licenses/by-nc-nd/4.0/). This content is not subject to CC BY 4.0.

Kong et al. developed biomimetic oxygen-carrying NPs and conjugated ultrasmall nanozyme on their surface; they further coated the NPs with bone marrow stromal cell membrane to target and successfully deplete leukemic cells in bone marrow and prevent homing of AML. Furthermore, the cell membrane acted as CXR4 antagonist to block the CXCR4/CXCL12-mediated homing of leukemia cells to the bone marrow and infiltration into other organs like the liver and spleen [136] (Figure 9).

**Figure 10:**
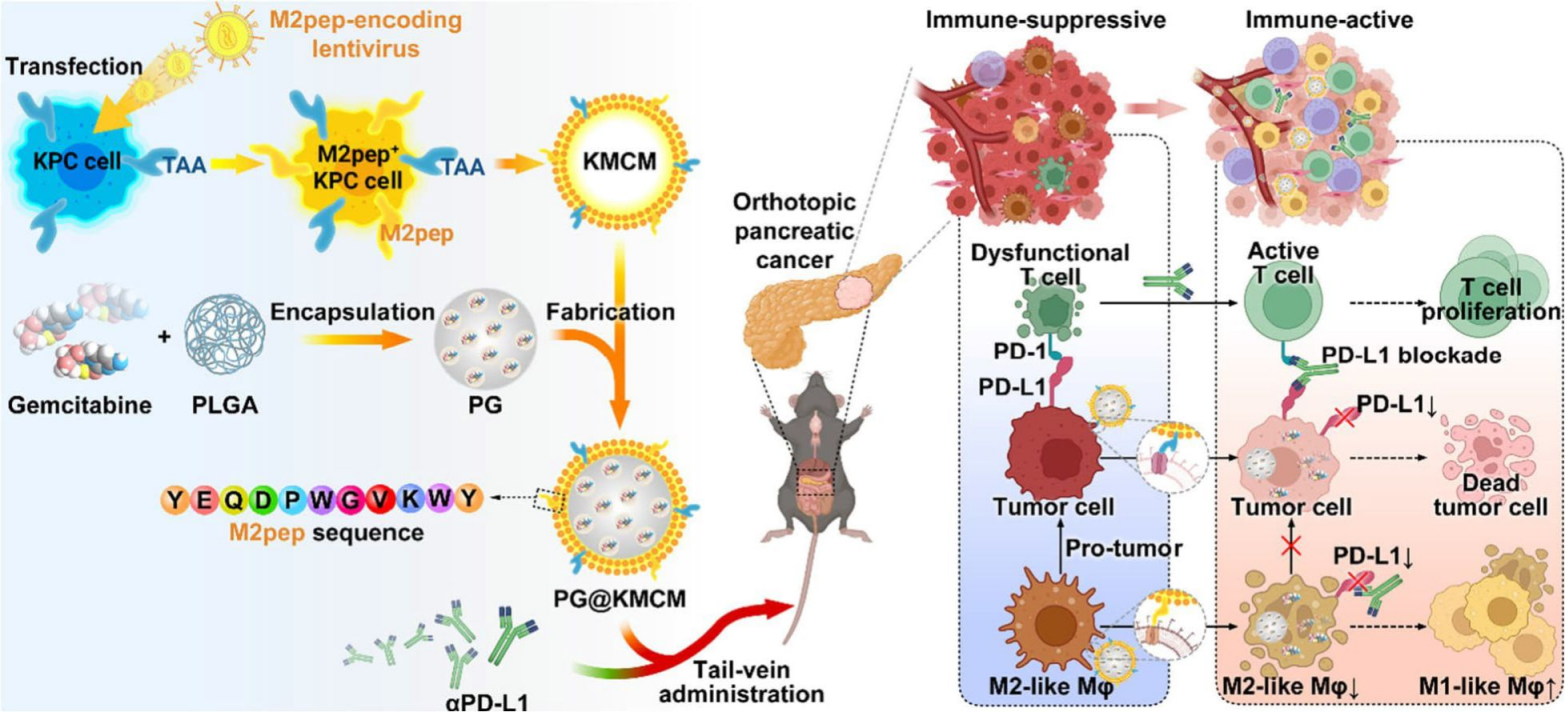
Fabrication of dual targeting PG@KMCM for chemoimmunotherapy in pancreatic cancer. Figure 10 was reproduced from [138] (© 2022 Wang et al., published by BMC (part of Springer Nature), distributed under the terms of the Creative Commons Attribution 4.0 International License, https://creativecommons.org/licenses/by/4.0).

Ke et al. altered the tumor glucose supply and metabolic pathways by designing RGD-modified, RBC membrane-coated glucose oxidase (Gox) and DOX metal organic framework bioreactors (RGD-mGZD). The RGD-mGZD NPs preferentially targeted the tumor site consuming the intratumoral oxygen and glucose to starve the tumor cells. At the same time DOX was released by the decomposition of the metal organic framework in acidic TME to induce chemotherapeutic effects and synergistically kill the tumor cells [137]. The immunosuppressive TME considerably attenuated the chemotherapeutic effect of various anticancer drugs. Therefore, reprogramming of TME and targeting tumor-associated macrophages (TAMs) could be a promising strategy to enhance chemotherapeutic efficacy. In this respect, Wang et al. synthesized bioengineered cancer cell membrane-coated, gemcitabine-loaded PLGA dual targeting NPs (PG@KMCM) for pancreatic cancer treatment. The PG@KMCM efficiently delivered gemcitabine to pancreatic cancer cells and TAMs simultaneously and potentiated the antitumor effect. Moreover, in combination with PD-L1, the PG@KMCM reprogrammed the immunosuppressive TME by eliminating the PD-L1 macrophages and therefore downregulating PD-L1 expression [138] (Figure 10).

**Figure 11:**
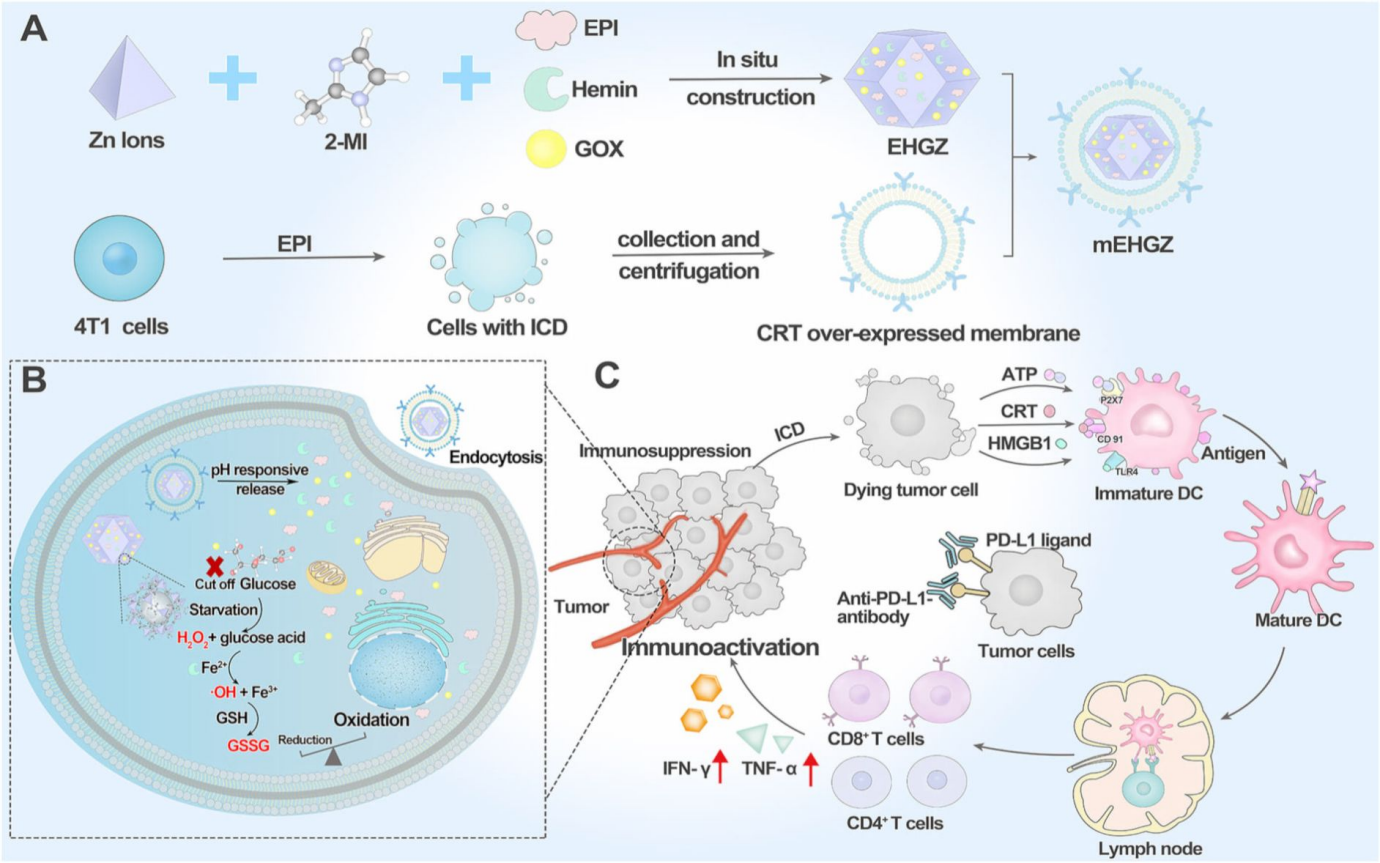
Self-amplified biomimetic nanosystem to induce ICD and activate an immune microenvironment to enhance the therapeutic effects of anti-PD-L1 antibody. (A) Preparation of CRT over-expressed tumor cell membrane coated EPI, Gox and hemin in ZIF-8 loaded mEHGZ NPs and (B) underlying mechanism (C) to create an immunosupportive microenvironment to boost the therapeutic effect of anti-PD-L1 antibody. Figure 11 was reproduced from [139] (© 2023 Z. Li et al., published by Elsevier B.V. on behalf of KeAi Communications Co. Ltd., distributed under the terms of the Creative Commons Attribution-NonCommercial-NoDerivatives 4.0 International License, https://creativecommons.org/licenses/by-nc-nd/4.0/). This content is not subject to CC BY 4.0.

Similarly, to attenuate the immunosuppressive TNBC TME, Li et al. constructed CRT-overexpressed tumor cell membrane-coated biomimetic NPs by encapsulating epirubicin (EPI), Gox, and hemin in ZIF-8 nanoparticles (mEHGZ). EPI induced immunogenic cell death (ICD), Gox and hemin initiated ROS generation, and the CRT membrane gave an “eat me” signal to dendritic cells (DCs) to invoke the tumor immunity cycle. Thus, the ICD effect promoted maturation of DCs and increased the infiltration of cytotoxic T lymphocytes at the tumor site, thus, reversing the immunosuppressive TME. Notably, mEHGZ in combination with anti-PD-L1 antibody dramatically reduced tumor growth and lung metastasis [139] (Figure 11).

**Figure 12:**
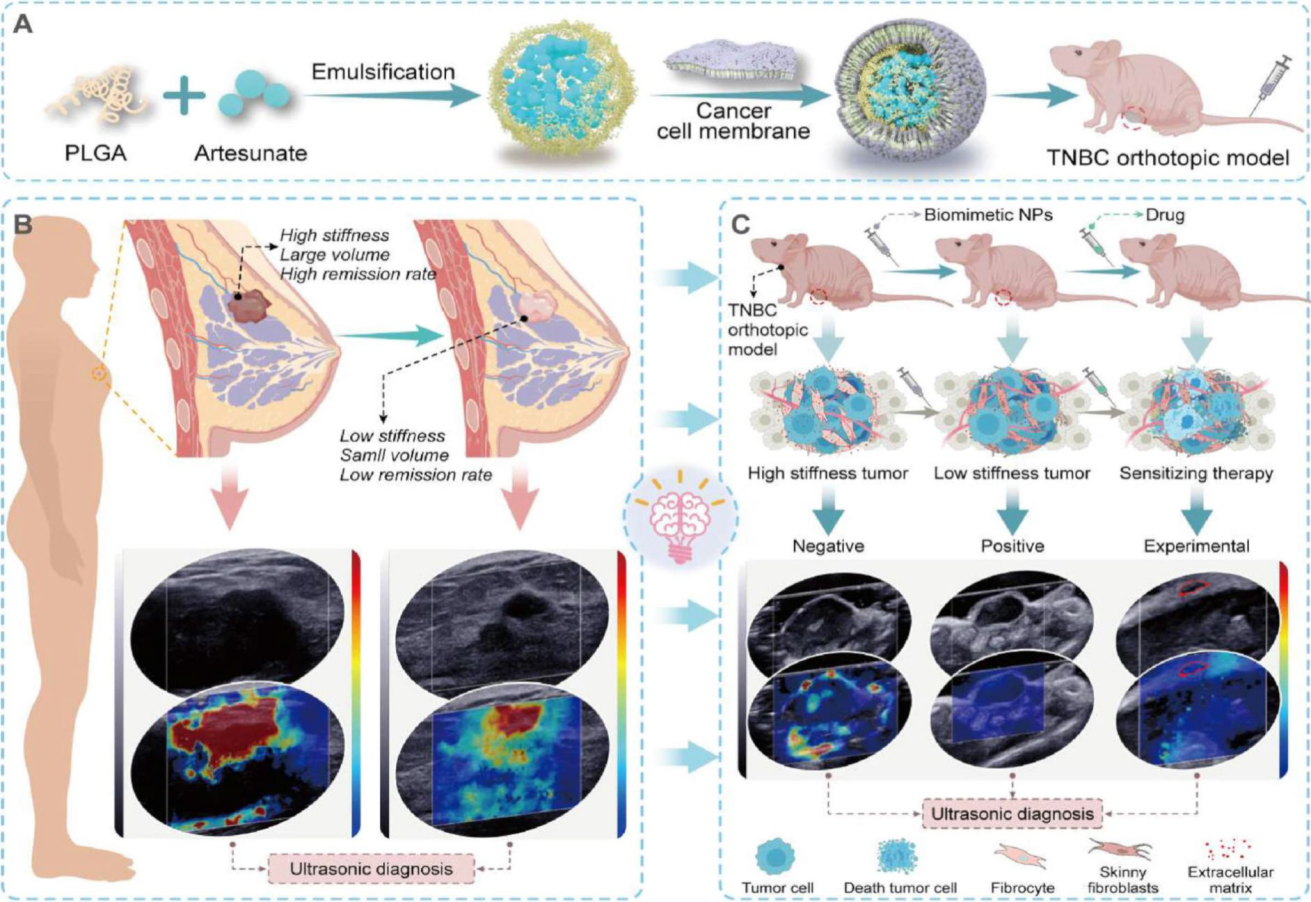
Schematic illustration of (A) fabrication of biomimetic NPs and (B, C) the mechanism by which shear wave elasticity imaging can predict TNBC treatment efficacy in MDA-MB-231 and E0771 orthotopic tumor models. Figure 12 was reproduced from [143] (© 2023 D. Zheng et al., published by Elsevier B. V. on behalf of KeAi Communications Co. Ltd.), distributed under the terms of the Creative Commons Attribution 4.0 International License, https://creativecommons.org/licenses/by/4.0).

To increase the chemotherapeutic drug delivery, Zhao et al. combined magnetite and DOX encapsulated in 6 BSA subunit to design a BSA magnetite nanotorpedo (BMNT). The BMNT significantly stops the leakage of DOX and prolonged its half-life in blood circulation to achieve efficient antitumor efficacy [140]. Against glioma, Du et al. designed hydrazone bond-conjugated DOX-manganese dioxide (MnO_2_) NPs coated with C6 cell membrane (MnO_2_-DOX-C6). MnO_2_ promoted the decomposition of H_2_O_2_ to produce oxygen and increase the ROS via a Fenton-like reaction. Interestingly, the C6 membrane coating allowed MnO_2_-DOX to target the glioma cells by homologous targeting and enhanced glioma cells apoptosis [141]. To improve the antitumor efficacy and bioavailability of chemotherapeutics, Gao et al. exploited albumin-mediated transportation and developed a biomimetic prodrug by modifying camptothecin with different fatty acid chain lengths. The series of in vitro and in vivo experiments confirmed the potent antitumor efficacy of CPT18C-HAS without obvious adverse effects [142]. It was reported that TNBC patients showed higher shear wave elasticity (SWE) α-SMA expression in tumor tissues than non-TNBC patients, which directly correlates to the neoadjuvant treatment efficacy. To confirm this phenomenon, Zheng et al. constructed TNBC membrane-coated, artesunate-loaded PLGA NPs (231M-ARS@PLGA) to regulate the SWE stiffness via cancer-associated fibroblast (CAF) inhibition. In the MDA-MB 231 and E0771 orthotopic tumor models, 231M-ARS@PLGA reduced the SWE stiffness and tumor hypoxia, which potently enhanced the antitumor effects of OTX and PD1 inhibitor. Importantly, single-cell sequencing demonstrated two main CAFs (extracellular matrix and wound-healing CAFs) that produce extracellular matrix could influence the tumor SWE stiffness as well as the antitumor effect of drugs. Moreover, biomimetic NPs reduced the CAF status, which in turn attenuates tumor hypoxia by increasing inflammatory blood vessels and oxygen transport capacity. This confirmed the role of CAFs on SWE stiffness and antitumor efficacy, which could be employed in clinical theranostics through non-invasive SWE imaging (Figure 12) [143].

#### 2.2 Gene therapy

**Figure 13:**
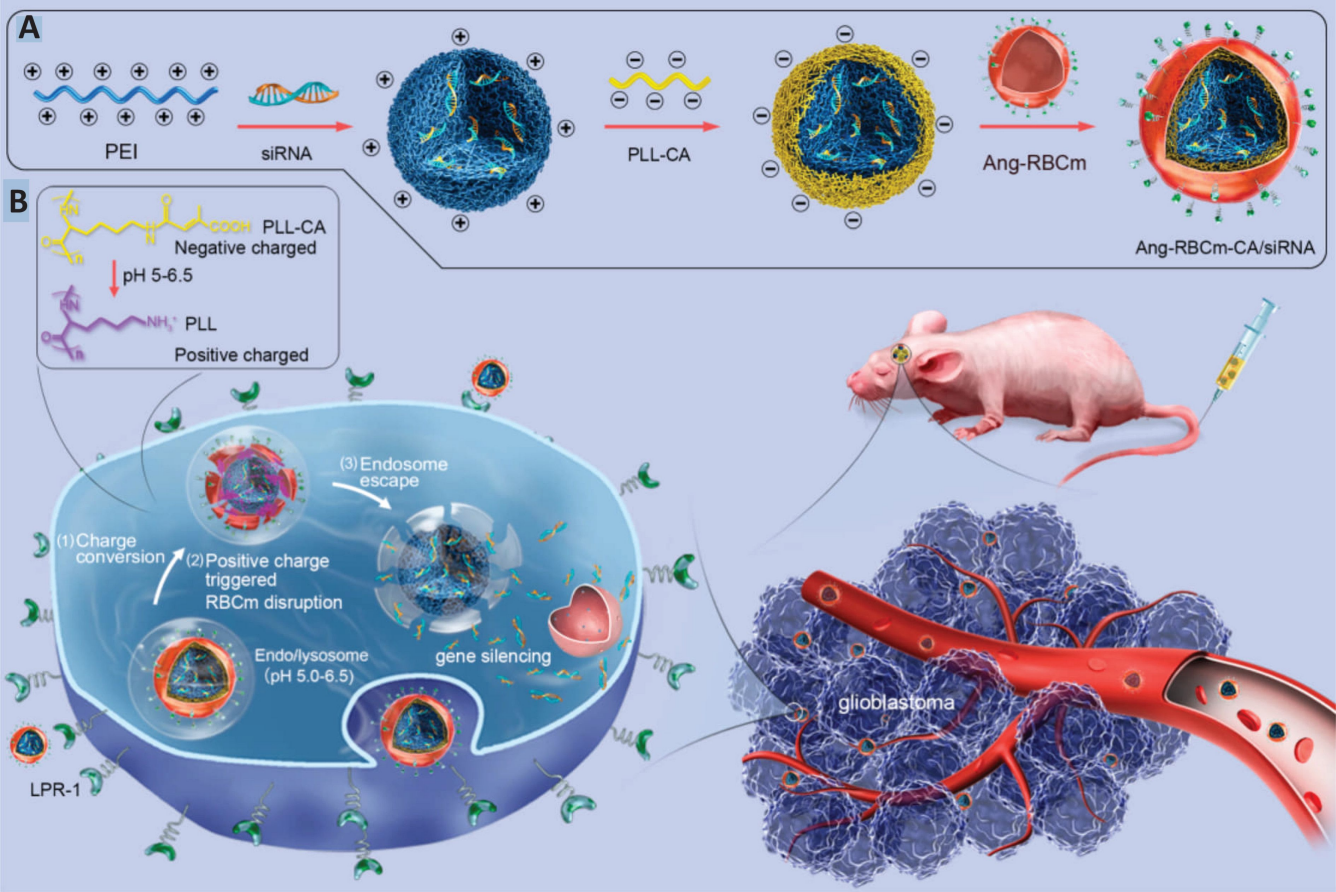
(A) preparation of Arg-RBCm-CA/siRNA and (B) pH-dependent siRNA release mechanism and overcoming BBB. Figure 13 was reprinted with permission from [149], Copyright 2020 American Chemical Society. This content is not subject to CC BY 4.0.

In order to achieve efficient delivery, the drug carriers must not degrade or be identified by nucleases and immune cells. Also, they need to pass multiple biological barriers and achieve high accumulation at the disease site with maximum internalization and endosomal escape [144]. In a recent study, Walweel et al. engineered a novel cationic star copolymer to deliver LC3 siRNA efficiently into TNBC cells. The polycationic structure provided strong electrostatic condensation with siRNA and enabled proton sponge-mediated endosomal escape, ensuring cytoplasmic release of the gene cargo. These LC3siRNA-loaded nanoparticles (LC3siRNA-NPs) exhibited pH-responsive behavior, enhanced cellular uptake, and potent suppression of autophagy. In vitro and in vivo experiments demonstrated that co-administration with doxorubicin significantly inhibited tumor growth and induced apoptosis, highlighting the system’s promise for overcoming chemoresistance in TNBC [145]. The success of efficient drug delivery to achieve considerable therapeutic outcomes highly depends on various critical structural parameters including charge, shape, and size [146]. Importantly, various problems exist associated with exogenous siRNA/genes, including easy degradation, short circulation time, immune clearance, low accumulation, and inability to penetrate the target cell membrane [147–148]. Gene therapy paves new ways in the treatment of incurable diseases by effective gene regulation strategies, such as a three-layer core–shell biomimetic nanostructure fabricated to overcome limitations in siRNA delivery to glioblastoma (Figure 13). The three-layer shell consisted of polyethyleneimine (PEI)-siRNA complex in the core, citraconic anhydride grafted poly-ʟ-lysine (PLL-CA) in the middle, and an outer layer of angiopep-2-decorated RBC membrane (Ang-RBCm). The RBC membrane allowed for longer circulation of NPs, without being recognized by the immune system. While further modification of the membrane with angiopep-2 provided enhanced BBB penetration. The inner PLL-CA cavity provided charge conversion in the acidic compartments of the tumor by proton sponge effects, resulting in NP escape from the acidic endosomes. Meanwhile, the outer RBC membrane shell is disrupted and accelerates the release of siRNA into the cytoplasm [149–150]. In contrast, Li et al. synergistically delivered a miR-144/451a cluster by constructing chitosan NPs (CAs) camouflaged with macrophage membranes for oral squamous cell carcinoma (OSCC) treatment. It was demonstrated that CAs coloaded with miR-144-MEXO/CA-miR-451a NPs significantly reduced the viability, migration, and invasion of OSCC cells; also, they substantially reduced the CAB39 and MIF expression in OSCC cells [151].

The targeted pH-responsive biomimetic nanodelivery system displayed high therapeutic efficacy with minimal side effects in orthotopic human glioblastoma-bearing animals. In one study, a worm-like nanostructure coated with RBC membrane for siRNA delivery has also been demonstrated. Herein, RBC membrane was modified with RGD peptide to achieve active targeting ability and a pH-dependent charge conversion strategy was involved to accomplish effective siRNA release [152]. In another example of RBC camouflaged particle, amphiphilic copolymer of PLGA-PEI was coated with RBC membrane to transfect endothelial cells with plasmid DNA for EGFR expression [153]. In another approach, CCM-based biomimetic nanomedicine for siRNA delivery has also been reported [154]. CCM-coated poly(β-amino ester) was successfully applied for siRNA delivery, resulting in cancer cell targeting, inhibition of PKL1 gene expression, and the induction of apoptosis in cancer cells. Besides the membrane coating strategy, a DNA nanotechnology-based nanosystem, which is inspired by holliday junction molecules, was demonstrated [155]. DNA tiles mimicking the holliday junction molecule structure were conjugated with gold nanoparticles and successfully used for the delivery of a neutral antisense oligonucleotide, morpholino, for silencing HER2 and ERα genes in breast cancer.

Within gene therapy applications, bioinspired tumor-homing nanomedicines have also been demonstrated for combinational therapies. Macrophage–CCM hybrid-coated PLGA was used to deliver siRNA against fibrinogen-like protein 1 and metformin, a metabolic immunomodulator for both gene therapy and immunosuppressive tumor microenvironment [156]. Other combinational therapy strategies using biomimetic nanoplatforms were reported with co-delivery of nucleic acid drugs and chemotherapeutics [157–159], and combinational gene therapy and photothermal therapy [160]. Recently, Liang et al. developed novel stealth and MMP2-activated biomimetic nanoparticles by using MMP2-responsive peptides to bind with miR-126-3p and then further camouflaged with RBC membrane (REMAIN). Due to the natural RBC membrane, REMAIN showed extended circulation, low toxicity, better biocompatibility, and immune escape; they induced the apoptosis of lung cancer cells, in vitro and in vivo [161].

Furthermore, Yang et al, inspired by human bone marrow mesenchymal stem cells, developed a biomimetic zeolitic imidazolate framework-8 to navigate herpes simplex virus type I thymidine kinase-encoded plasmids and ganciclovir for lung cancer treatment. The biomimetic NPs showed long circulation and enhanced tumor accumulation, and significantly inhibited the tumor. These biological bomb structures killed the transfected as well surrounding lung cancer cells by a “bystander effect” and efficiently suppressed lung cancer [162].

#### 2.3 Photothermal/photodynamic therapy

Photothermal therapy (PTT), a new class of cancer treatment that uses heat absorbed by light-absorbing materials, is a non-invasive method with certain advantages, including reduced toxicity and strong anti-tumor efficacy [163–165]. The working principle of PTT is based on the conversion of absorbed light energy into heat, resulting in photoablation, which leads to cell damage and death [166]. Overall, PTT presents great potential in improving recovery time and better outcomes in cancer treatment [163,167]. To enhance the therapeutic efficiency of PTT, biomimetic NPs have been developed by camouflaging the PTT particles with different cell membranes to allow them to be used in cancer diagnosis and treatment. The biomimetic PTT NPs diffuse into tumor tissues and are engulfed by the cancer cells; then light is applied to heat up the tissue by using PTT, destroying cancer cells. Previously, RBC membrane coating increased and improved the effect of PTT by prolonging blood circulation and enabling precise tumor homing. It was observed that RBC membrane-coated NPs significantly improve PTT effects and reduce tumor with 100% survival up to 45 days [168–170].

In another study, Wu et al. employed “disassembly–reassembly” technology to obtain reconstituted RBCs (rRBCs) for a membrane-coated delivery platform. It was found that the rRBC membrane improved stability, circulation time, and immune escape after removing the endogenous proteins and lipids on the membrane. After synthesizing biomimetic rRBC membrane, the IR780 (NIR) fluorescence dye was loaded as a photosensitizer for PTT to generate biomimetic “IR780-rRBC” NPs. The antitumor effect of IR780-rRBC was also evaluated. After laser treatment, the tumor temperatures in mice treated with IR780-rRBC and IR780-rRBC NPs increased up to 60 and 70 °C, respectively. Importantly, following photothermal therapy in R780-RBC and IR780-rRBC groups, a significant necrosis in the tumor and a noticeable suppression in the tumor volume were observed [171]. Li et al. developed hybrid nanovesicles (TT3-oCB NP-EXOs) with enhanced second near-infrared (NIR-II, 900–1700 nm) fluorescence and PTT, consisting of tumor cell-derived exosomes (EXO) and TT3-oCB nanoparticles. The TT3-oCB NP-EXOs showed promising and stable photothermal conversion capacity under 808 nm irradiation to be used as biomimetic NPs for NIR-II fluorescence imaging-guided PTT of tumors. The TT3-oCB NP-EXOs demonstrated prolonged blood circulation and enhanced tumor uptake in vitro and in vivo [172].

In addition to the direct ablation of tumor cells, photothermal therapy also elicits immune responses, which could be used to treat metastatic tumors by producing tumor-associated antigens [173–174]. Therefore, combining PTT and immunotherapy is thought to be ideal for efficient anticancer immunotherapy [175–177]. Recently, inorganic photothermal transducing agents coated with antibodies/peptides have been successfully applied for combined cancer therapy. This biomimetic nanosystem comprised FePSe_3_ modified with chitosan, CT26 cancer cell membrane (CCM), and anti-PD-1 peptide (APP) for PTT-immunotherapy. To improve the immunotherapeutic effects, the anti-PD-1 peptide was covalently bound to the chitosan-stabilized FePSe_3_ nanosheets. Under NIR laser irradiation, the photothermal effects produced by FePSe_3_-APP-CCM not only killed cancer cells but also induced intense immune responses both in vitro and in vivo [175].

In another report, Wei et al. developed a cancer cell membrane-coated homotypic targeting gold nanocage, m@Au-D/B NCs, loaded with DOX and l-buthionine sulfoximine (BSO) for promising anticancer combination therapy by eliciting ferroptosis and immune responses. DOX and BSO induced ferroptosis by glutathione consumption and ROS generation. Moreover, the gold nanocages evoked PTT and photochemical catalysis, generating more ROS under NIR irradiation. At the same time, the m@Au-D/B NCs-associated PTT and ROS generation could repolarize TAMs and initiate the release of proinflammatory cytokines, as well as significantly inhibit tumor growth at minimum toxicity [178]. In order to downregulate heat shock protein (HSP) expression, Shu et al. constructed 4T1 cell membrane-coated biocompatible mesoporous Prussian blue nanoparticles (PBLM@CCM NPs) loaded with lonidamine (LN) and ᴅʟ-menthol. The PBLM@CCM NPs selectively delivered LN to reduce HSP and overcome heat endurance in PTT by inhibiting intracellular ATP production. Furthermore, the PBLM@CCM NPs allowed for photoacoustic imaging and produced heat to promote the phase change of ᴅʟ-menthol for ultrasound imaging [179].

**Figure 14:**
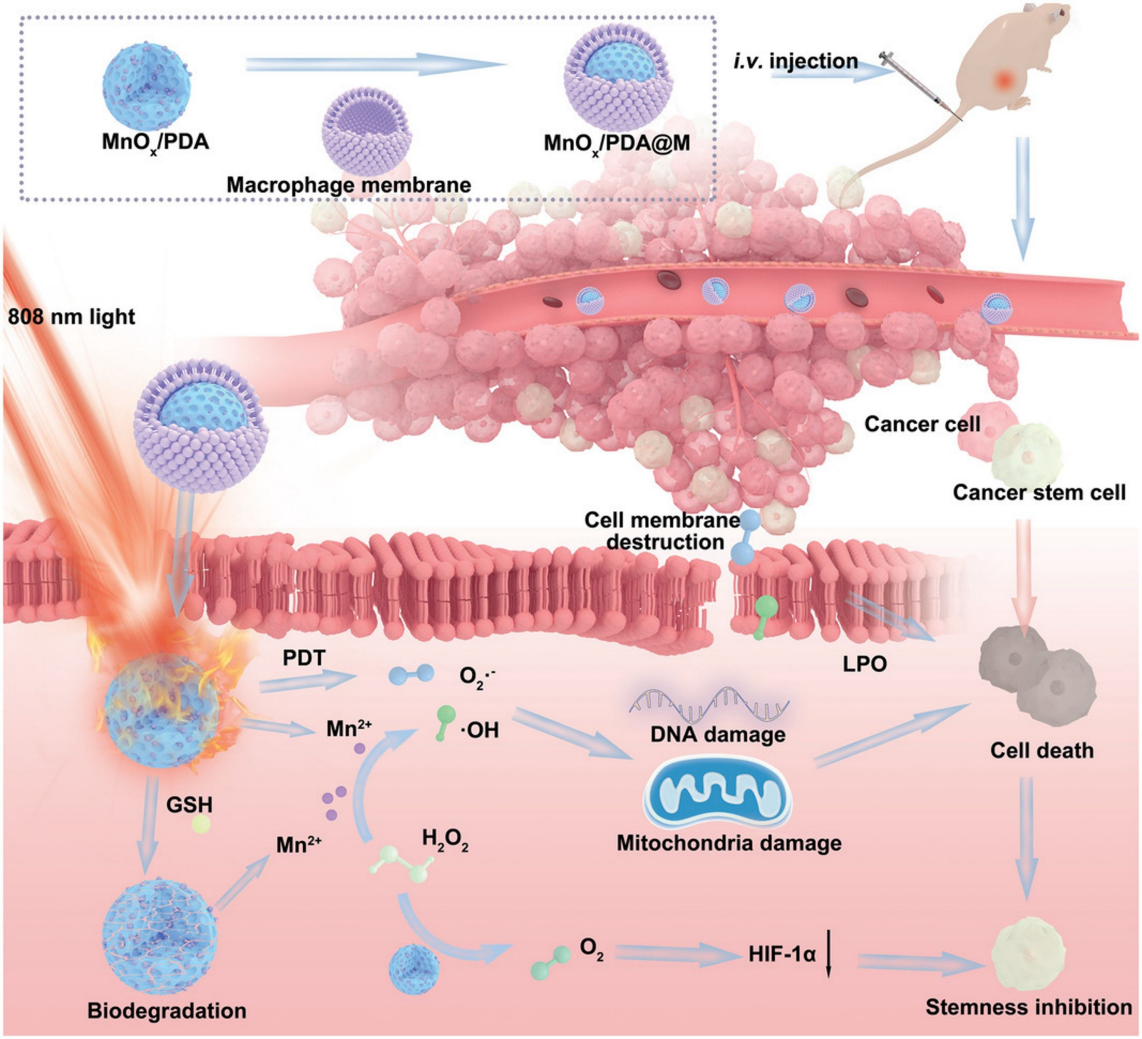
Macrophage membrane-coated MnO*_x_*/PDA nanobombs for antitumor therapy with chemodynamic, photodynamic, photothermal, and biodegradation properties. Figure 14 was reproduced from [180], S. Liu et al., “Biomimetic Nanobomb for Synergistic Therapy with Inhibition of Cancer Stem Cells”, *Small*, with permission from John Wiley and Sons. Copyright © 2022 Wiley-VCH GmbH. This content is not subject to CC BY 4.0.

It was observed that cancer stem cells (CSCs) are the key to cancer metastasis, recurrence, and chemotherapeutic resistance. To inhibit CSCs, Liu et al. designed MnO*_x_*-loaded polydopamine (MnO*_x_*/PDA) nanobombs with chemodynamic, photodynamic, photothermal, and biodegradation properties. The MnO*_x_*/PDA nanobombs directly target the CSCs in the TME by generating hyperthermia, hypoxia, and oxidative stress. Furthermore, macrophage membrane-coated MnO*_x_*/PDA targeted and synergistically inhibited tumor volume up to 70.8% in colorectal cancer tumor model [180], as shown in Figure 14.

**Figure 15:**
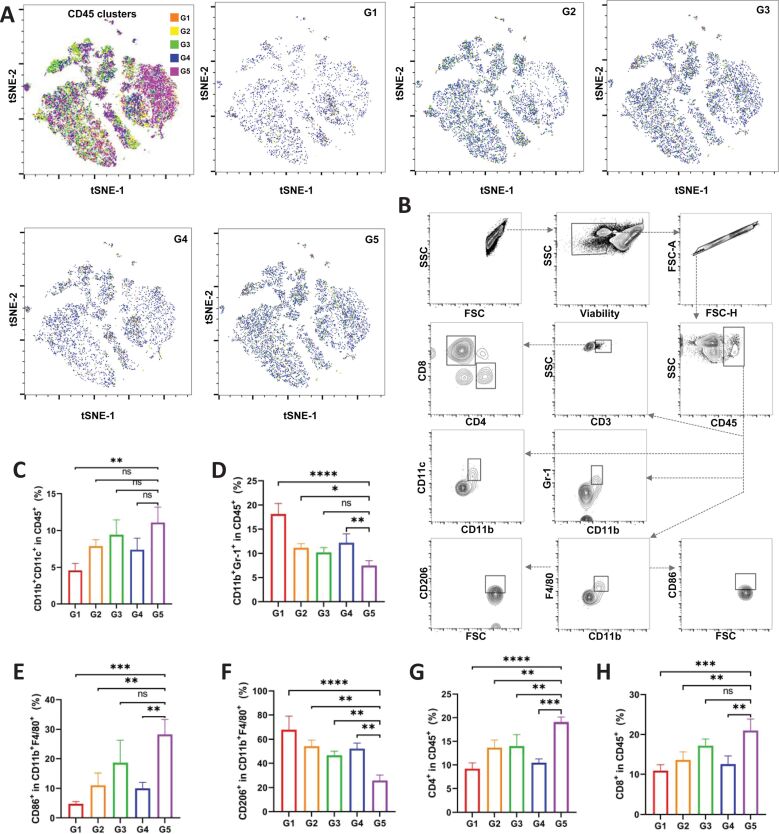
Myeloid-derived suppressor cell membrane-coated camouflage gold nanorods covered with silica and manganese dioxide (GNRs@SiO_2_@MnO_2_@MDSCs, GSMM) and TME after different treatments. (A) Mass cytometry of CD45+ tumor-infiltrating leukocytes. The t-distribution stochastic neighbor embedding (t-SNE) plot of CD45+ tumor-infiltrating leukocytes of a total 25 samples (pooled data) (left panel: overlaid with color-coded clusters) and density t-SNE plots of CD45+ tumor-infiltrating leukocytes in different treatment groups (G1: PBS, G2: PBS + laser, G3: GSM + laser, G4: GSMM, G5: GSMM + laser). (Laser irradiation was conducted at 4 h post-injection of nanoparticles with a 1064 nm laser for consecutive 5 min, 0.6 W/cm^2^). (B) Schematic gating strategy for the identification of various immune cell populations in a B16F10 xenograft model. Flow cytometric quantification of (C) CD11b+CD11c+ dendritic cells, (D) CD11b+Gr-1+ MDSCs, (E) CD86+CD11b+F4/80+ M1 macrophages, (F) CD206+CD11b+F4/80+ M2 macrophages, and (G) CD4+ T cells, H) CD8+ T cells in tumor tissues. Figure 15 was reproduced from [181] (© 2023 Y. Zhao et al., published by Elsevier B.V. on behalf of KeAi Communications Co. Ltd., distributed under the terms of the Creative Commons Attribution-NonCommercial-NoDerivatives 4.0 International License, https://creativecommons.org/licenses/by-nc-nd/4.0/). This content is not subject to CC BY 4.0.

In terms of antitumor immunotherapy, the immunosuppressive TME and inferior drug targeting are major challenges. To address these, Zhao et al. developed myeloid-derived suppressor cell (MDSC) membrane-coated camouflage gold nanorods covered with silica dioxide and then coated them with manganese dioxide (MnO_2_) to obtain GNRs@SiO_2_@MnO_2_@MDSCs (GSMM). GSMM actively targeted MDSCs and the localized surface plasmon resonance of GNRs developed in the NIR-II window via the MnO_2_ layer coating, conveying NIR-II photothermal and photoacoustic imaging to GSMM; also Mn^2+^ release could be used for magnetic resonance imaging. The Mn^2+^ catalyzed the reaction of H_2_O_2_ into ·OH for chemodynamic therapy (CDT) to activate the cGAS-STING mechanism and the secretion of interferon type I, proinflammatory cytokines, and chemokines. Furthermore, the PDT and CDT-mediated ICD of tumor cells further enhanced the antitumor immunity via exposure of CRT, HMGB1, and ATP [181], as shown in Figure 15.

**Figure 16:**
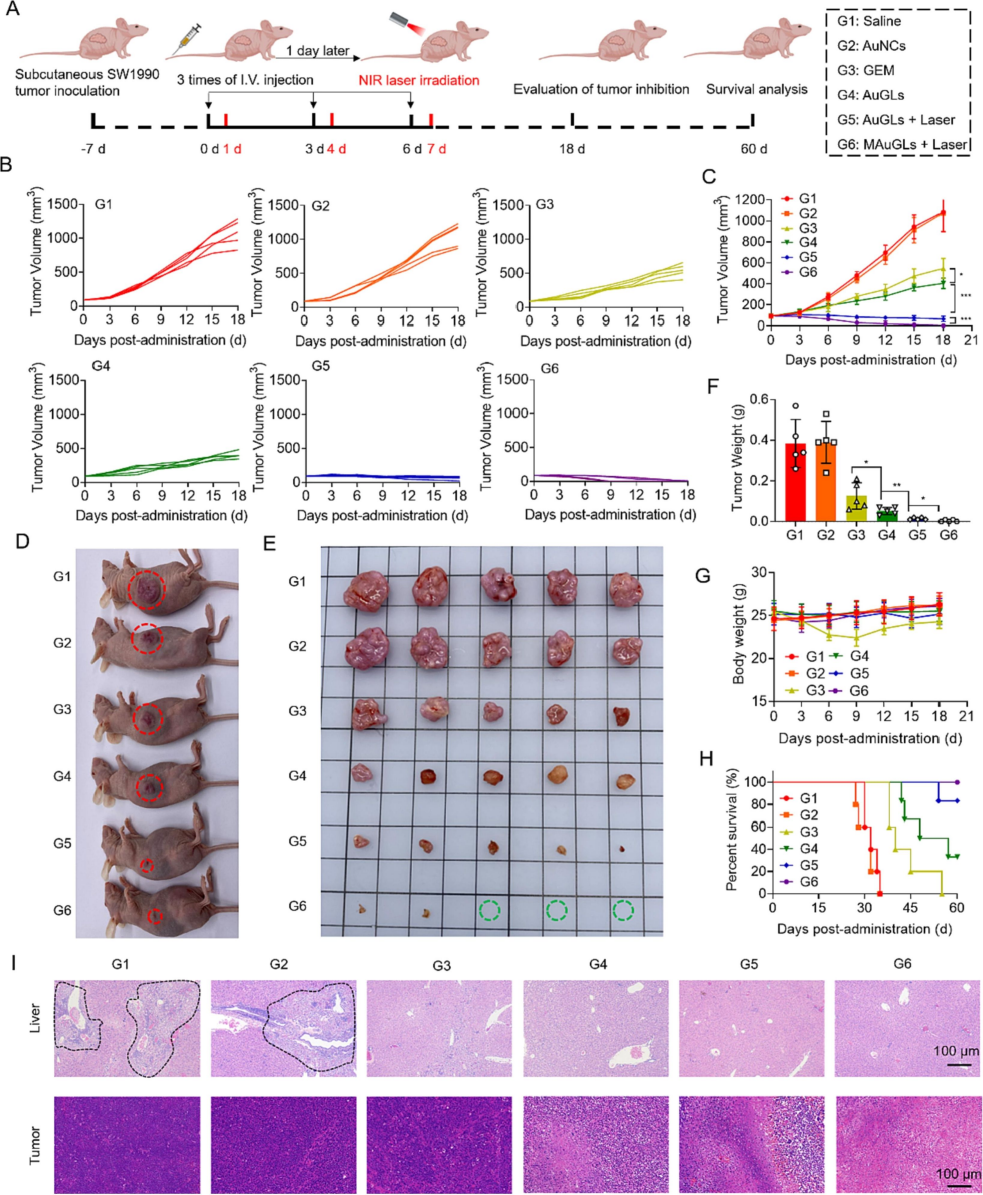
Antitumor efficacy of TME responsive PDAC cell line camouflaged gold nanocages. (A) Schematic illustration of the design of the experiments, (B, C) relative tumor growth curves until day 18, (D) image of the tumor-bearing mice on day 18, (E) excised tumors on day 18, (F) weights of excised tumors on day 18, (G) changes in body weights of tumor-bearing mice within 18 days, (H) survival graph up to 60 days, and (I) H&E staining of livers and tumors of the mice. The black dashed lines indicated the metastatic tumors in the livers. Figure 16 was reprinted from [185], *Journal of Controlled Release*, vol. 354, by F. Zhang; Q. Hu; B. Li; Y. Huang; M. Wang; S. Shao; H. Tang; Z. Yao; Y. Ping; T. Liang, “A biomimetic nanodrug for enhanced chemotherapy of pancreatic tumors“, Pages 835–850, Copyright (2023), with permission from Elsevier. This content is not subject to CC BY 4.0.

The inter- and intra-individual heterogeneities of tumors and TME and the differences between heterotopic and orthotopic tumors limit the translational potential of nanoplatforms in accurate tumor therapy [182–183]. To address this Wu et al., used head and neck squamous cell carcinoma (HNSCC) cell membranes and patient-derived cell membranes to surface-modify gold NPs (Au@C-CCM or Au@C NP) and evaluated the antitumor efficacy in subcutaneous xenograft, tongue orthotopic xenograft, immune competent primary and distal tumor models, as well as patient-derived xenograft models. It was observed that Au@C-CCM produced up to 44.2% photothermal conversion for primary HNSCC therapy, inducing immunotherapy to inhibit tumor metastasis via photothermal therapy-mediated ICD. Furthermore, due to the homologous targeting mechanism, the homologous CCM-coated NPs demonstrated distinct tumor ablation in all four models, indicating efficient antitumor therapy [184]. Regarding the most malignant and high-MDR pancreatic ductal adenocarcinoma (PDAC), Zhang et al. designed TME-responsive PDAC cell line-camouflaged gold nanocages (AuNCs) to simultaneously deliver the chemotherapeutic GEM and a nitrogen oxide (NO) donor (ʟ-Arg) for efficient tumor homing and reduced chemoresistance. The high intracellular level of glutathione cleaved the disulfide bond and triggered the release of GEM. Also, the elevated ROS level activated ʟ-Arg and generated NO, facilitating GEM to penetrate PDAC tumor tissues by vasodilation and normalization of blood vessels to induce potent antitumor effects. Importantly, the AuNCs not only served as a photothermal agent but also generated significant photoacoustic signals to monitor drug accumulation and distribution inside the tumor cells [185], as shown in Figure 16.

Photodynamic therapy (PDT) is one of the most important therapy types that can be used alone or in combination with other traditional treatment methods in cancer treatment [186]. Compared to traditional cancer treatment methods, PDT is considered to be very advantageous due to its very low intervention level. The process of producing reactive oxygen species is based on various biochemical reactions that occur when light-sensitive agents, called photosensitizers, interact with light of the appropriate wavelength [187]. Photosensitive agents transfer this absorbed energy to the molecules in the living microenvironment through two different types of mechanisms. In type-1 reactions, the high-energy photosensitizer can directly react with an electron-donating substrate to form superoxide anion radicals. In type-2 reactions, the energy of the high-energy photosensitizer is transferred directly to ground state oxygen ^3^O_2_ in the environment, producing highly reactive singlet oxygen ^1^O_2_. The production of singlet oxygen and superoxide anions using any of these two mechanisms create a toxic effect on cells through damaging lipids, proteins, and nucleic acids. Accordingly, cell death and cell destruction result [188–191].

To date, numerous NPs have been studied to increase the concentration of photosensitizers and to ensure their accumulation at the tumor site. At the same time, studies on liposomes, dendrimers, silver, and gold nanoparticles have been carried out to improve the phototoxic properties of photosensitizers. PDT is applied to cancer types characterized by no therapeutic efficacy or lacking effective therapeutic targets. In a study conducted by Zhang et al., PDT, PTT, and the anticancer agent DOX have been applied in combination on TNBC, and a novel biomimetic platform has been developed with leukocyte-platelet hybrid membrane and dendritic large pore mesoporous silicon nanoparticles, LPHM and DLMSNs, respectively. IR780 and DOX have been co-loaded into the DLMSNs to carry DLMSNs-DOX-IR780 (DDI) NPs. The leukocyte/platelet hybrid membrane was coated with DDI NPs to prepare the final biomimetic platform, LPHM-DDI NPs. These NPs showed superior TNBC targeting ability and very high PTT/PDT performance in vitro and in vivo. After NIR laser irradiation with the synergistic effect of PDT and PTT, cytotoxicity was observed in TNBC cells after treatment with LPHM-DDI NPs. Furthermore, tumor growth and recurrence were effectively suppressed in the TNBC mouse model. This suggests that PTT/PDT provides a promising biomimetic nanoplatform as a combination therapy against TNBC [192]. In a similar study, platelet-mimetic NPs have been used to create more effective PDT therapy. Van Straten et al. developed platelet-mimetic PLGA NPs encapsulating verteporfin as the photosensitizer, which shifted the absorption peak from 682 to 712 nm, allowing for better absorption in deeper tissues. Thus, platelet-mimetic PLGA NPs achieved higher accumulation in tumor tissues in comparison with control and RBC membrane-coated NPs. Furthermore, the platelet-mimetic PLGA NPs exhibited significant tumor ablation without causing any side effects under NIR irradiation [187].

Recently, PDT received great attention due to its ability to induce ICD. In this respect, Wu et al. developed multifunctional MPCO biomimetic NPs (4T1Mem@PGA-Ce6/Ola) to codeliver the photosensitizer chlorin e6 (Ce6) and olaparib (Ola) with the function of preventing DNA repair. The nanoplatform demonstrated efficient tumor homing, and Ce6 and Ola induced synergistic antitumor efficacy under laser irradiation. Furthermore, MPCO activated the cyclic guanosine monophosphate–adenosine monophosphate synthase–interferon gene stimulator signaling (cGAS-STING) pathway to produce cytokines. The damage-associated molecular patterns induced by ICD can work with these cytokines to recruit and stimulate the maturation of dendritic cells and induce a systemic anti-tumor immune response [193].

#### 2.4 Sonodynamic therapy

Sonodynamic therapy (SDT) is a non-invasive therapeutic strategy that enables tumor cells to be killed by activation of photosensitive compounds [194]. It is also an anti-cancer method working through localized light transmission derived from PDT. The combination of PTT and SDT shows great promise for synergistic antitumor therapy. In SDT, the tumor site is exposed to focused ultrasound (FUS), which offers improved tissue penetration and reduces potential off-target effects [195]. Deep tissue penetration of SDT can overcome the inherent deficiency of PTT in targeting deeper tumors. In some applications, FUS may offer a wider variety of applications regarding focus and tumor coverage. Similar to photosensitizers, FUS activates “sonosensitizers” that selectively accumulate in tumor cells and generate ROS. In addition, SDT can be integrated with different FUS approaches. For example, FUS can be administered i.v. to permeabilize cell membranes and increase drug delivery through transient openings in the blood–brain or blood–tumor barriers. Injected contrast agents can be placed [176]. Moreover, the accumulation of sonosensitizers in tumor cells can be enriched with enhanced permeability and retention (EPR) effect.

**Figure 17:**
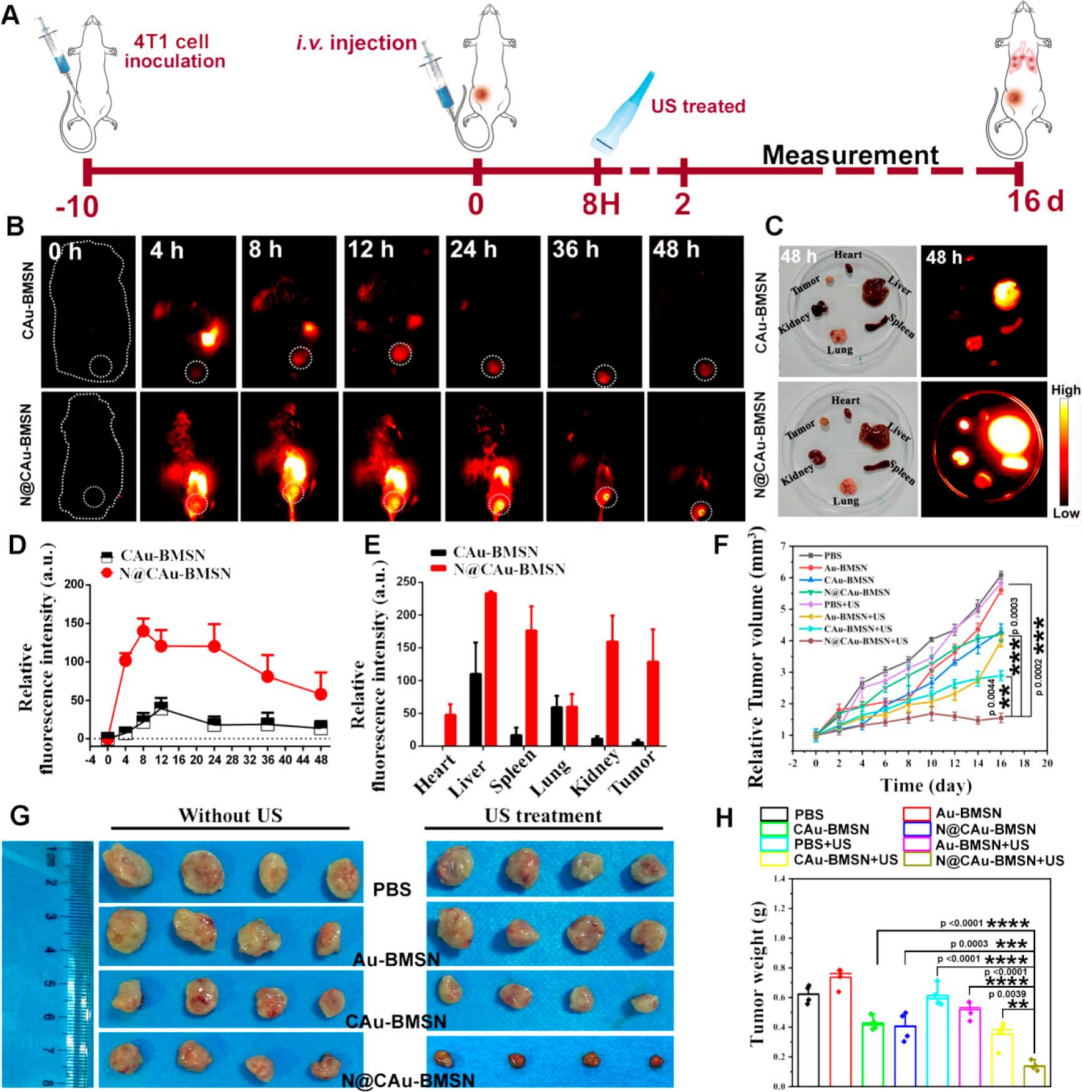
(A) Schematic representation of an in vivo study of N@CAu-BMSNs, (B) NIR-II ﬂuorescence imaging of ICG-labeled CAu-BMSNs or N@CAu-BMSNs at diﬀerent time points after i.v. injection, (C) ex vivo biodistribution in tumor and major organs isolated from 4T1-tumor-bearing mice after 48 h of ICG-labeled CAu-BMSN or N@CAu-BMSN injection, respectively, (D, E) ﬂuorescence intensity of tumor and major organs after intravenous injection at diﬀerent time points, (F) tumor volume change of mice after receiving US treatment, (G) tumor images, and (H) average tumor weight of mice after US treatments. Figure 17 was reprinted with permission from [196], Copyright 2020 American Chemical Society. This content is not subject to CC BY 4.0.

Zhang et al. took advantage of the ability of the macrophage membrane coating to target the tumor and avoid the RES. As shown in Figure 17, compared to ICG-labeled CAu-BMSNs, the ICG-labeled N@CAu-BMSNs showed a significantly high NIR-II fluorescence signal in tumors, confirming active targeting and RES evasion of the macrophage membrane. Also, the macrophage membrane-coated biomimetic nanosystem demonstrated an excellent synergistic antitumor effect of SDT/CO treatment to effectively suppress tumor growth, relapse, and metastasis to the lung. The strategy based on sonodynamic/CO therapy and IDO signal inhibition is a promising approach against tumor recurrence and lung metastasis in clinical trials [196].

**Figure 18:**
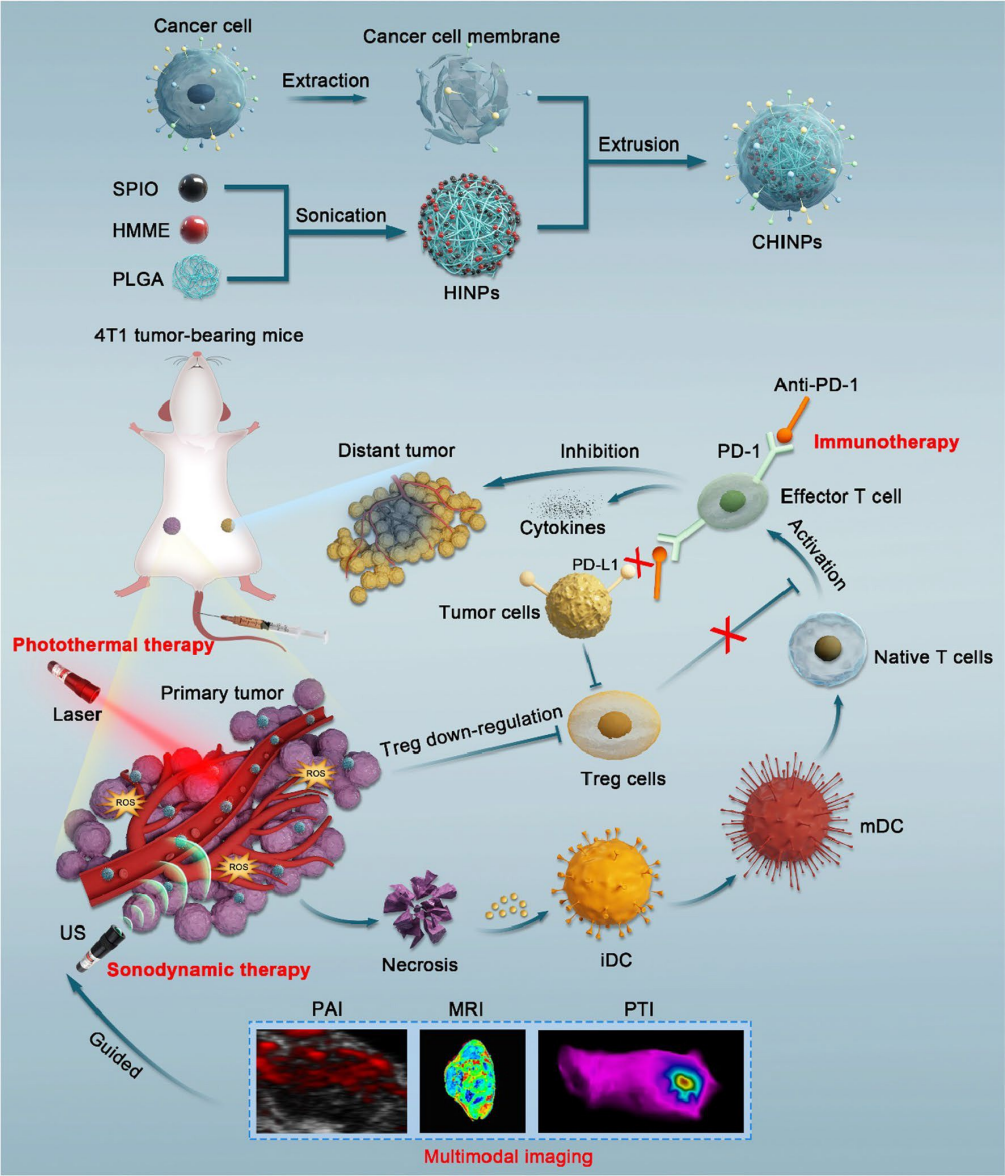
Illustration of CHINPs synthesis and combined effects of PTT/SDT enhanced anti-PD-1 against primary and distant tumor. Figure 18 was reproduced by [197] (© 2022 X. Lin et al., published by BMC (part of Springer Nature), distributed under the terms of the Creative Commons Attribution 4.0 International License, https://creativecommons.org/licenses/by/4.0).

In another study, a triple therapy combining PTT, SDT, and anti-PD-1 immunotherapy, guided by multimodal imaging to treat 4T1 tumors in mice model (Figure 18) [197]. The authors used PLGA-based biomimetic nanoparticles (CHINPs) coated with 4T1 cancer cell membranes and loaded with ultrasound- and laser-responsive agents (HMME and SPIO). These biomimetic nanoparticles actively targeted homologous 4T1 tumors, enabled precise tumor targeting, and eliminated primary tumors by multiple imaging-guided PTT/SDT treatment, exposing tumor antigens, and enhancing immune responses by boosting CD8^+^ T cells and reducing Tregs. Anti-PD-1 antibodies further strengthened T cell activity by blocking immune checkpoints, leading to improved tumor control and reduced metastasis.

Aydin et al. developed a novel FUS-responsive biomimetic nanoparticle system by coating gold nanocones (AuNCs) with B16F10 melanoma cell membranes (CCM@AuNCs). These nanoparticles exhibited strong homologous targeting toward melanoma cells, enabling enhanced accumulation and might facilitate mechanical ablation upon therapeutic FUS exposure. Following activation, this approach may promote the release of tumor-associated antigens and potentially enhance immune cell infiltration into the TME. Such a strategy might represent a promising platform for melanoma-targeted FUS-assisted immunotherapy [198].

**Figure 19:**
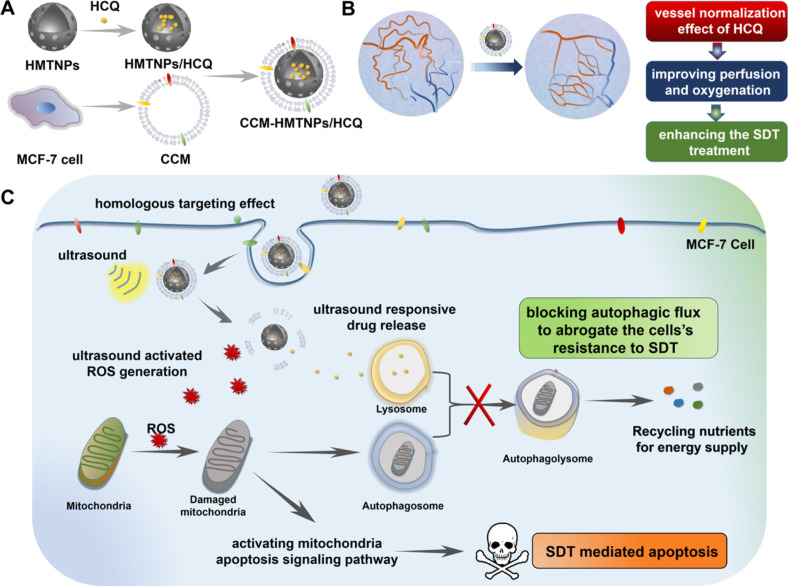
SDT mechanism of CCM-HMTNPs/HCQ via autophagy regulation in breast cancer treatment. Figure 19 was reprinted with permission from [199], Copyright 2019 American Chemical Society. This content is not subject to CC BY 4.0.

Another biomimetic nanoplatform study concerned the resistance of cancer cells to SDT. Feng et al. have introduced a biomimetic nanoplatform based on hollow mesoporous titanium dioxide nanoparticles (HMTNPs) with autophagy inhibitor (hydroxychloroquine sulfate, HCQ) loading and cancer cell membrane (CCM) coating (Figure 19) [199]. Due to the functionalization of the biomimetic surface, CCM-HMTNPs/HCQ are able to evade macrophage phagocytosis and actively recognize tumors with their homologous targeting ability. Subsequently, HCQ released in response to ultrasound stimulation has the ability to block autophagic flux and cut off the nutrient supply derived from damaged organelles, which is expected to abolish the cells’ resistance to SDT. Thus, the study demonstrated a new therapeutic route to target autophagy in SDT.

**Table 1:** Summary of practical benefits and challenges of biomimetic nanoparticles in comparison to conventional nanoparticles.

Aspect	Biomimetic nanoparticles	Conventional nanoparticles

immune evasion	* mimic natural cell membranes	
targeting specificity	* high target specificity via cell membrane proteins and natural ligands	
circulation time	* long retention time mimicking natural cells (e.g., RBC membrane)	
biocompatibility and toxicity	* improved biocompatibility	
drug loading and therapeutic efficacy	* ability to load various cargos, including drugs and siRNA	* good drug payload and solubility
manufacturing complexity	* complex preparation of cell membranes and coating steps	
stability and storage	* biological materials may degrade during storage	* longer shelf life under standard conditions
safety/immunogenicity risk	* dependent on cell source and patient variability	* known side effect profiles, particularly for approved drugs
clinical translation	* limited progression to clinical usage despite promising preclinical results	* some clinically approved nanoparticle therapies (e.g., nab-paclitaxel)
regulatory approval	* complex classification and limited precedent	* familiar to regulatory bodies with established frameworks

## Conclusion and Future Perspectives

Our comprehensive review has underscored the remarkable potential of biomimetic NPs in the realm of drug delivery. These biomimetic NPs exhibit a unique amalgamation of distinct biochemical functionalities, heightened biocompatibility, augmented bioavailability through evasion of immune responses, and precise targeting, thereby limiting premature clearance en route to designated sites of action. Also, by integrating or fabricating biomaterials onto the surface of nanoparticles, they mimic the biological features and functions of native cells. Therefore, it is evident that biomimetic NPs represent a promising avenue for clinical translation.

However, the deployment of biomimetic NPs in clinical applications is not devoid of formidable challenges that warrant meticulous attention. The issues surrounding the selection of the most appropriate cell membrane type and source, the scalability and reproducibility of manufacturing processes, post-fabrication sterilization methods, long-term stability and storage conditions, the development of personalized biomimetic NPs, concerns related to cross-reactivity, regulatory approval hurdles, and the economic feasibility of large-scale production must be considered.

We anticipate that sustained efforts in the advancement of cutting-edge technologies and a concerted approach to address these multifaceted challenges will herald a transformative paradigm shift in the field of novel drug delivery systems. Such advancements hold the promise of revolutionizing the way therapeutics are designed, formulated, and administered, ultimately enhancing the efficacy and precision of drug delivery and thus benefiting both scientific and medical communities alike.

## Data Availability

Data sharing is not applicable as no new data was generated or analyzed in this study.
